# Basket trials in oncology: a systematic review of practices and methods, comparative analysis of innovative methods, and an appraisal of a missed opportunity

**DOI:** 10.3389/fonc.2023.1266286

**Published:** 2023-11-14

**Authors:** Adetayo Kasim, Nathan Bean, Sarah Jo Hendriksen, Tai-Tsang Chen, Helen Zhou, Matthew A. Psioda

**Affiliations:** ^1^ Disease Area Strategy, Oncology Biostatistics, GlaxoSmithKline, Brentford, United Kingdom; ^2^ Statistics and Data Science – Innovation Hub, GlaxoSmithKline, Philadelphia, PA, United States; ^3^ Medical and Market Access, Oncology Biostatistics, GlaxoSmithKline, Stevenage, United Kingdom; ^4^ Disease Area Strategy, Oncology Biostatistics, GlaxoSmithKline, Philadelphia, PA, United States

**Keywords:** basket trials, information borrowing, Bayesian model averaging, tumor-agnostic treatment effect, tumor types

## Abstract

**Background:**

Basket trials are increasingly used in oncology drug development for early signal detection, accelerated tumor-agnostic approvals, and prioritization of promising tumor types in selected patients with the same mutation or biomarker. Participants are grouped into so-called baskets according to tumor type, allowing investigators to identify tumors with promising responses to treatment for further study. However, it remains a question as to whether and how much the adoption of basket trial designs in oncology have translated into patient benefits, increased pace and scale of clinical development, and de-risking of downstream confirmatory trials.

**Methods:**

Innovation in basket trial design and analysis includes methods that borrow information across tumor types to increase the quality of statistical inference within each tumor type. We build on the existing systematic reviews of basket trials in oncology to discuss the current practices and landscape. We conceptually illustrate recent innovative methods for basket trials, with application to actual data from recently completed basket trials. We explore and discuss the extent to which innovative basket trials can be used to de-risk future trials through their ability to aid prioritization of promising tumor types for subsequent clinical development.

**Results:**

We found increasing adoption of basket trial design in oncology, but largely in the design of single-arm phase II trials with a very low adoption of innovative statistical methods. Furthermore, the current practice of basket trial design, which does not consider its impact on the clinical development plan, may lead to a missed opportunity in improving the probability of success of a future trial. Gating phase II with a phase Ib basket trial reduced the size of phase II trials, and losses in the probability of success as a result of not using innovative methods may not be recoverable by running a larger phase II trial.

**Conclusion:**

Innovative basket trial methods can reduce the size of early phase clinical trials, with sustained improvement in the probability of success of the clinical development plan. We need to do more as a community to improve the adoption of these methods.

## 1 Introduction

Advancement in genomics technology has enabled innovation in oncology drug development over the last decade. A growing adoption of a precision medicine, with the aim to identify and develop effective targeted therapies, characterizes the current landscape of pharmaceutical drug development. It requires that a new treatment must not only address a disease defined by the histology and anatomical site from which it arose, but also the specific molecular, genetic, or immunologic subtype (1). To date, the U.S. Food and Drug Administration (FDA) has approved six tumor-agnostic therapies (2): selpercatinib was approved in September 2022 for patients with locally advanced or metastatic rearranged during transfection (RET) fusion-positive solid tumors (3); dabrafenib was approved in June 2022 in combination with trametinib for patients with unresectable or metastatic solid tumors with BRAF V600E mutation (4); dostarlimab, a programmed cell death protein 1 (PD-1) inhibitor, was approved in August 2021 for adult patients with mismatch repair deficient recurrent or advanced endometrial cancer (5); entrectinib, a tyrosine kinase inhibitor (TKI) was approved in August 2019 for adults and pediatric patients 12 years of age and older with solid tumors that have a neurotrophic tyrosine receptor kinase (NTRK) gene fusion without a known acquired resistance mutation (6); pembrolizumab, a PD-1 inhibitor, received accelerated approval in 2017 for adult and pediatric patients who have unresectable or metastatic solid tumors microsatellite instability-high (MSI-H) or deficient mismatch repair (dMMR) (7); larotrectinib, a tropomyosin kinase receptor (TRK) inhibitor, was approved in 2017 for adult and pediatric patients with unresectable or metastatic solid tumors with neurotrophic TRK fusion (8).

Innovation in biotechnology and clinical trials, matched with advanced computational tools, holds promise to accelerate the discovery and development of new targeted therapies. One example of methodological innovation is the design of master protocols, or trials that simultaneously evaluate the effect of multiple investigational drugs and/or multiple cancer types under a single overarching protocol (9). Master protocols in oncology allow detection of specific signal pathways strongly associated with driver gene mutations, cancer cell growth, and progression (10). A basket trial is a particular type of master protocol that evaluates the efficacy and safety of a targeted therapy in multiple diseases that share a common molecular alteration (11) or a tumor agnostic effect. Basket trials can also be used to identify the tumor types where the drug is active with a single operationally efficient homogeneous protocol. Of the six tumor-agnostic therapies approved by the FDA, two development programs—selpercatinib (NCT03157128) and dabrafenib (NCT02465060)—used a basket trial.

A recent systematic review of basket trial master protocols identified a large increase in the number of basket trials in the past 14 years, from 1 basket trial in 2009 to 49 trials in 2019 (11). However, it remains a question whether increasing adoption of basket trials has translated into patient benefit, either through higher response rates because of precision treatment or because of increasing options for rare tumor types that are less represented in oncology drug development (12). There are also challenges on how to evaluate treatment efficacy in basket trials, arising from the disconnect between the *implied* homogeneity of responses (based on the expectation of a tumor-agnostic effect based on one common molecular alteration) and the heterogeneity *observed* between different tumor types included in basket trials. Statistical methods have been developed in recent years to address these challenges, and can be classified broadly into tumor-specific analysis, pruning-and-pooling methods, Bayesian hierarchical modeling approaches, and model averaging methods. In a process referred to as *information borrowing*, many of the proposed statistical methods allow for data characterizing the effect of a therapy in one tumor type to inform to some degree the effect in a different—but possibly similar—tumor type. This can increase the amount of information available for performing inferences, such as testing whether each tumor-specific treatment effect exceeds some context-specific threshold. Despite the statistical advantages of these innovative information borrowing methods, their uptake has been slow in the design and analysis of basket trials (13).

To make innovative basket trial methods more accessible to the clinical and research community, we conduct and present a systematic review designed to understand current basked trial practice in oncology and identify barriers to using these methods more often. We also performed a review of recently proposed statistical methodology with a focus on conceptual understanding of the methodologies rather than an in-depth review of mathematical and statistical details. For the clinical community to adopt these advanced statistical methods, we believe it is critical to build a conceptual understanding of the ideas in sufficient depth to engage in meaningful discussion with statisticians during basket trial planning stages. Through case studies, we illustrate the performance of advanced information borrowing methods using actual data from completed basket trials. We also provide a glimpse into the future of basket trials in oncology by showing how a basket trial might be used as a strategic component of a hypothetical clinical development plan (CDP) that includes phase Ib and phase II oncology trials.

## 2 Methods

The systematic literature reviews of basket trial practice and basket trial methods were done in accordance with the Preferred Reporting Items for Systematic Reviews and Meta-Analysis (PRISMA) guidelines (14). The EQUATOR checklist for the systematic review is provided in **Supplementary Tables S4 in Supplementary File 1**.

### 2.1 Data sources and searches

Systematic searches were conducted on February 20, 2023, in MEDLINE, Embase, and the Cochrane Central Register of Controlled Trials. The search strategy mirrored the approach of Park et al. (11), with minor modifications, such as modifying search terms to focus only on basket trials, and we supplemented the search with a review of bibliographies from included publications and trial registries (ClinicalTrials.gov) for registered basket trial protocols. Further details on the number of hits from each database are presented in **Supplementary Table S1–S3 in Supplementary File 1**.

### 2.2 Study inclusion and exclusion criteria

In addition to the trials already identified by Park et al. (11), we searched for any unique basket trials referenced in abstracts and papers from January 2019 through February 2023 for a systematic review of basket trial practice. The search for basket trial methods was from January 2001 to February 2023. **Table 1** provides inclusion and exclusion criteria as per PICOS (population, intervention, comparator, outcomes, study design). The eligible abstracts and papers were restricted to English language only.

**Table 1:** PICOS criteria.

Category	Inclusion Criteria
Population	Humans
Interventions	No restrictions
Comparator	No restrictions
Outcomes	No restrictions
Study design	Basket trial design
Other	Peer-reviewed publications and conference abstracts with results or published protocols in the English language

Three reviewers (SJH, NB, and AK) independently reviewed all abstracts identified in the literature searches and assessed their eligibility. SJH and NB identified individual basket trials and extracted their key characteristics from the eligible abstracts, the corresponding full-text publications, bibliographies of published literatures, protocols, and trial registries. Discrepancies in trial selection were resolved by discussion with a third investigator (AK) or the wider team including MP and HZ.

### 2.3 Data extraction

Abstracts were reviewed to assess whether the studies meet the eligibility criteria for basket trial practice or basket trial methods. Basket trial practice includes any publication that reports on the design and findings from a basket trial, and papers for basket trial methods were defined as any paper that proposed innovative statistical methodology for basket trial design and/or analysis. The full texts were reviewed, and further decisions were made whether to extract data for each paper or not. SJH, NB, and AK independently screened the abstracts, reviewed full texts, and extracted data using bespoke data extraction templates developed by the team.

### 2.4 Data synthesis

We describe the basket trial practices in oncology using a narrative and descriptive statistics. No meta-analysis was done. Further information about the method is presented in **Supplementary File 1**.

## 3 Results

We identified 468 unique abstracts from our database searches. From these and our search on ClinicalTrials.gov, we identified 234 trials which warranted an in-depth review based on the inclusion and exclusion criteria. In total, 146 trials met our inclusion criteria for the review of basket trial practice—138 trials had ClinicalTrials.gov NCT numbers whereas 8 trials were not registered in ClinicalTrials.gov. For the methods search, 41 publications met our inclusion criteria for the review of statistical methods for the design and analysis of basket trials. The PRISMA diagrams for the basket trial practice and the basket trial methods systematic reviews are provided in **Supplementary Figures S1, S2 in Supplementary File 1**, and a complete list of trials and methodological papers are provided in **Supplementary Files 2, 3**, respectively.

### 3.1 Review of basket trial practices

A systematic review of how master protocols are reported identified inconsistencies in how studies self-identified as a master protocol, with considerable variability in the definition of basket, umbrella, and platform trials (15). A systematic review of basket trials by Park et al. (11) discussed the landscape of basket trials with a focus on trends, trial and disease characteristics, and regional representations. Park et al. (16) presented an overview of precision oncology basket and umbrella trials for clinicians, with illustrations of basket trial and umbrella trial design with examples. Meyer et al. (17) reviewed the evolution of master protocol clinical trial designs, reporting that most basket trials had a binary primary endpoint, no control group, and analyses that used frequentist methods. A more recent systematic review by Haslam et al. (12) focused on basket trials in oncology with published results in which they characterized the correlation between the size of a basket and the incidence of the respective tumor. Another recent review by Haslam et al. (18) discussed the importance of tissue origin and molecular target, noting that differences in response rates depended on tumor type. For example, breast and ovarian cancers were likely to have higher response rates than sarcoma or head and neck cancers. Our current review of basket trial practices and methods complements these existing reviews while focusing on type of design and analytic framework used for analysis.

**Figure 1:**
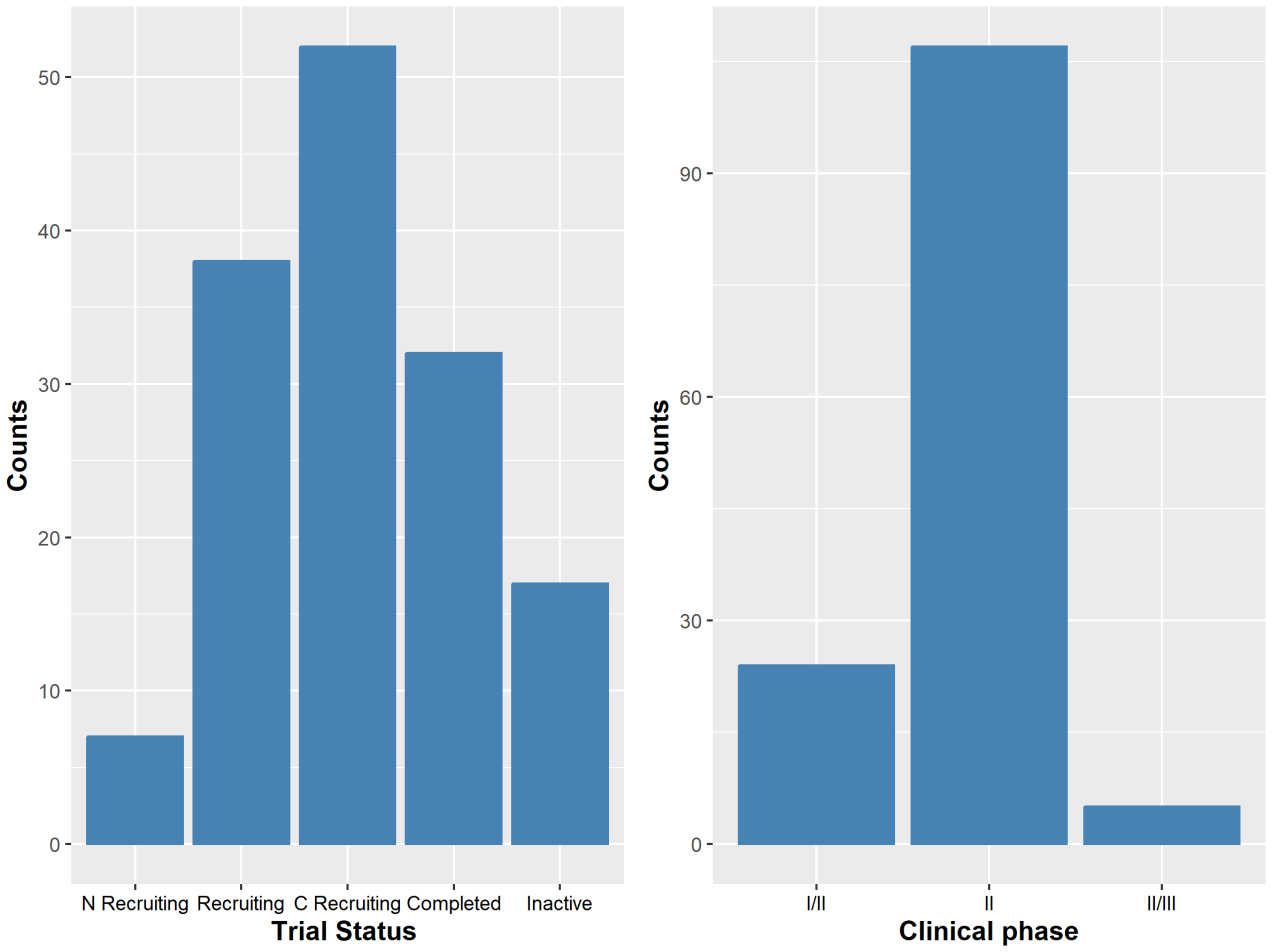
The left panel shows the number of trials by their status. ‘N Recruiting’ denoted trials that were not yet recruiting; ‘Recruiting’ are ongoing trials with active recruitment; ‘C Recruiting’ denotes active trial with recruitment closed; ‘Completed’ denotes completed trials; and ‘Inactive’ are trials that have been terminated, suspended, withdrawn or have unknown status. The right panel shows the number of trials by their clinical phase.

Of the 146 trials, 7 were not yet recruiting, 38 were ongoing/recruiting, 52 were active with closed recruitment, and 32 were completed; 17 trials had been terminated, withdrawn, or listed with unknown status (see **Figure 1**). We found that 75% (109/146) of trials investigated a monotherapy agent, while 25% (37/146) investigated a combination therapy. Most of the basket trials were in phase II (73%, 107/146), whereas 23% (34/146) of trials were in the dose expansion portion of a phase I study (i.e., phase Ib or phase I/II), and 3% (5/146) were designated as phase II/III (see **Figure 1**). Similar to Park et al. (11) and Haslam et al. (12), all phase Ib or phase II basket trials identified in this review were single-arm non-randomized open label studies, and 91% (133/146) of the trials had objective response rate as either a primary or secondary endpoint. Further information about the review of basket trials in practice and the risk of bias are presented in the **Supplementary File 1**.

#### 3.1.1 Risk of bias

To assess the risk of bias for each of the completed basket trials, we used the Risk of Bias In Non-randomized Studies of Interventions (ROBINS-I) tool developed for Cochrane systematic reviews (19). Due to common practices and standardization of the objective response rate (ORR, RECIST (20)) in the design of early-phase oncology trials, we do not discuss risks of bias that relate to the design of each trial (e.g., confounding, missing outcomes). Instead, we focus on risks of reporting bias associated with the publication of results. According to ROBINS-I, bias can occur when results for reporting are selected from (1) multiple outcome measurements within the outcome domain, (2) multiple analyses of the intervention-outcome relationship, or (3) different subgroups. The risks of bias from these first two sources are minimal in basket trials due to the defining of trial endpoints in the design stage and the specification of analysis methods prior to data analysis. However, bias can be introduced in the selection of which tumor-specific results are reported. Of the completed basket trials, approximately 38% (12/32) are subject to reporting bias with reasons including the reporting of only the pooled response rate (8/32), reporting results for only some tumor types (1/32), or not providing any publicly available results (3/32). Further, only 55% (16/29) of the completed trials on ClinicalTrial.gov had published results within the registry, whereas 38% (11/29) only had references to publications with results and 7% (2/29) do not provide results in any form. To reduce reporting bias and to increase transparency of trial results, we recommend that the results for each and all tumor types be published directly in ClinicalTrial.gov regardless of the strength of evidence supporting treatment efficacy.

### 3.2 Review of basket trial methods

An underlying assumption of statistical methods for basket trial design is that response to the targeted therapy is determined by a biomarker and is not heavily influenced by tumor histology (21), lending itself to a consideration that all tumor types in a basket trial share a similar response rate. As a result, tumor types are sometimes naïvely pooled together to estimate a single response rate during the final analysis of a basket trial. Such approach can increase the type I error rate (i.e., false positive/discovery rate) at the trial level. A tumor-level type I error rate is defined as the rate of falsely progressing a tumor type to the next phase of the clinical development when a treatment is not active on the tumor type (hereafter referred to as the tumor being an inactive tumor type), while the trial-level type I error rate is defined as the rate of falsely progressing any of the tumor types in a basket trial design when all tumor types are inactive.

We identified 41 methodological papers, of which 32% (13/41) use frequentist methods and 68% (28/41) use Bayesian methodology. Frequentist methods typically rely on p-values for hypothesis testing objectives (e.g., whether tumor-specific response rates exceed a pre-specified threshold), whereas Bayesian methods combine prior information and the trial results as part of a continual data stream in which inferences are updated each time new data become available (22). 5% (2/41) of papers proposed methods that incorporate independent analyses of data for each tumor type (23, 24) without borrowing information across tumor types. The remaining papers each propose a method that incorporates information borrowing, most of which can be grouped into one of three classes based on the information borrowing mechanism: pruning-and-pooling methods, Bayesian hierarchical models, and model averaging methods.

#### 3.2.1 Pruning-and-pooling methods

Of the proposed methods, 22% (9/41) suggest a two-stage design using a frequentist pruning-and-pooling approach. Under the most basic two-stage design, an interim analysis is performed at the first stage to determine which tumor types are active (i.e., treatment has an effect) and which are inactive. Enrollment in inactive tumor types is stopped, or “pruned”, and the active tumor types that passed the interim analysis are then pooled together to estimate the overall response rate or tumor-specific response rates. Proposed adaptions of the pruning-and-pooling approach include designs with any type of endpoint (25–28) or restricted to either a binary endpoint (29–32) or time-to-event endpoint (33). Only two pruning-and-pooling methods were published with publicly available software. Lack of software to implement the methods could hinder accessibility and ease of use by the wider community.

#### 3.2.2 Bayesian hierarchical models

**Figure 2:**
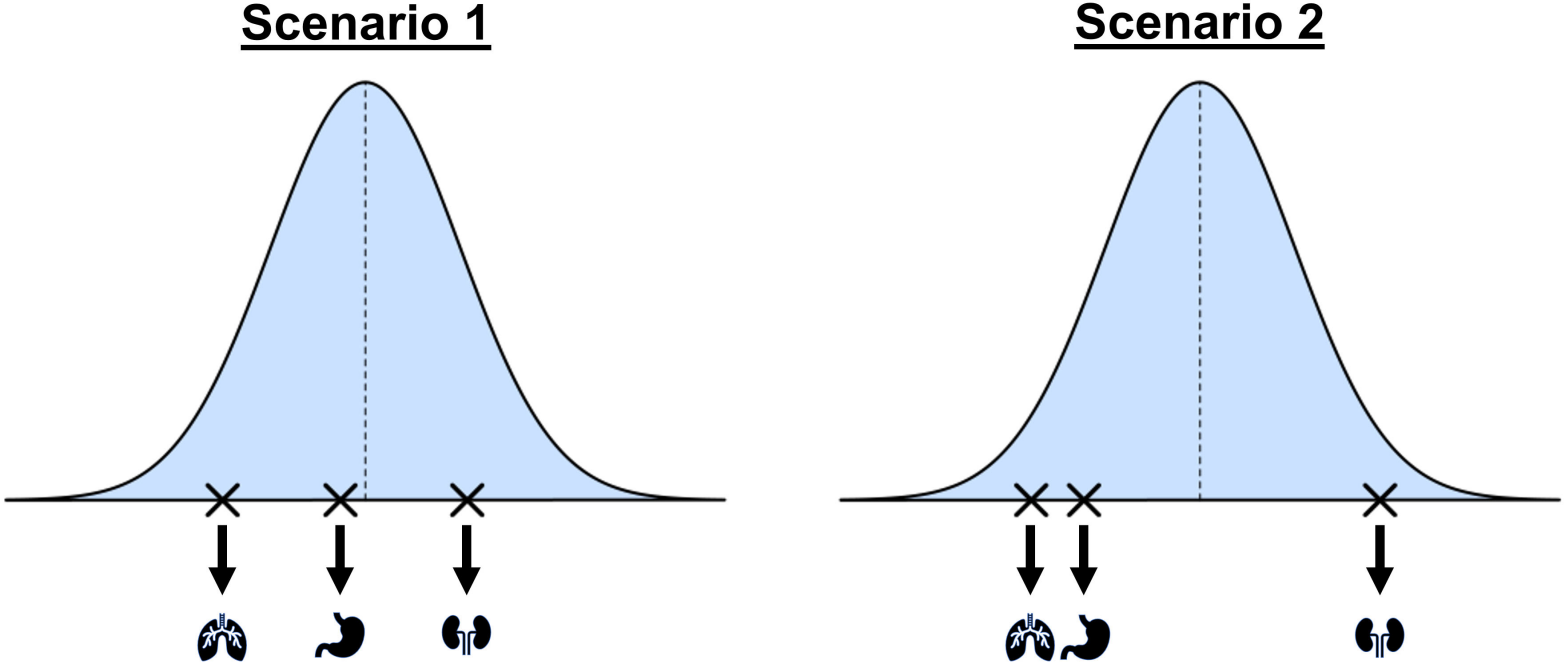
A conceptual illustration of a hierarchical model for the design and analysis of basket trials. The blue curve shows the assumed common distribution for treatment response rates. Scenario 1 assumes the tumor types are exchangeable and Scenario 2 shows a situation where response rates may be more similar for some tumor types than others.

A common method for borrowing information across different tumor types is a Bayesian hierarchical model (BHM), which assumes the response rates for all tumor types share a common underlying (bell-shaped) distribution as illustrated in **Figure 2**. Scenario 1 shows an example of a basket trial where response rates are from different locations of the bell-shape distribution without any obvious pattern. We refer to this as the *exchangeability assumption*, and we refer to the tumor types as being *exchangeable* with one another. However, the exchangeability assumption may not always be valid in practice. As an example, scenario 2 illustrates a case in which the response rates for two tumor types are more similar compared to the third tumor type.

Of the proposed methods, 39% (16/41) used a variation of the BHM. Thall et al. (34) first proposed the use of the BHM for trials with multiple disease types, and Berry et al. (35) extended this application to basket trials in oncology. Several methods relax the assumption of exchangeability in the BHM, including first testing for heterogeneity in the response and fitting a BHM only if the tumor types are deemed sufficiently homogeneous (36). Tumor types are assumed to be exchangeable with one another with some probability that can either be prespecified (37) or estimated by the data (38). Another approach is clustering similar tumor types into subgroups and then fitting a separate BHM within each cluster (39–43). Chu et al. (44) proposed a *calibrated* BHM (CBHM) which uses a fixed value for the between-tumor variance that is calculated via simulation studies to ensure the amount of borrowing is not substantial in the event that a large degree of heterogeneity is observed between tumor-specific response rates. Other variations of the BHM includes joint modeling of toxicity and efficacy (45), using a continuous biomarker to define subgroups of participants (46, 47), and conditioning information borrowing on the correlation between response rates (48).

#### 3.2.3 Model averaging methods

Of the 41 proposed methods, 12% (5/41) use model averaging to facilitate information borrowing across tumor types based on the general idea that different models are defined to represent unique scenarios of how the underlying tumor-specific response rates may relate to one another. Each model is fit to estimate the ORR for a subset of tumor types, and a weighted average of these model-specific results is then calculated to obtain the overall ORR for each tumor type.

**Figure 3:**
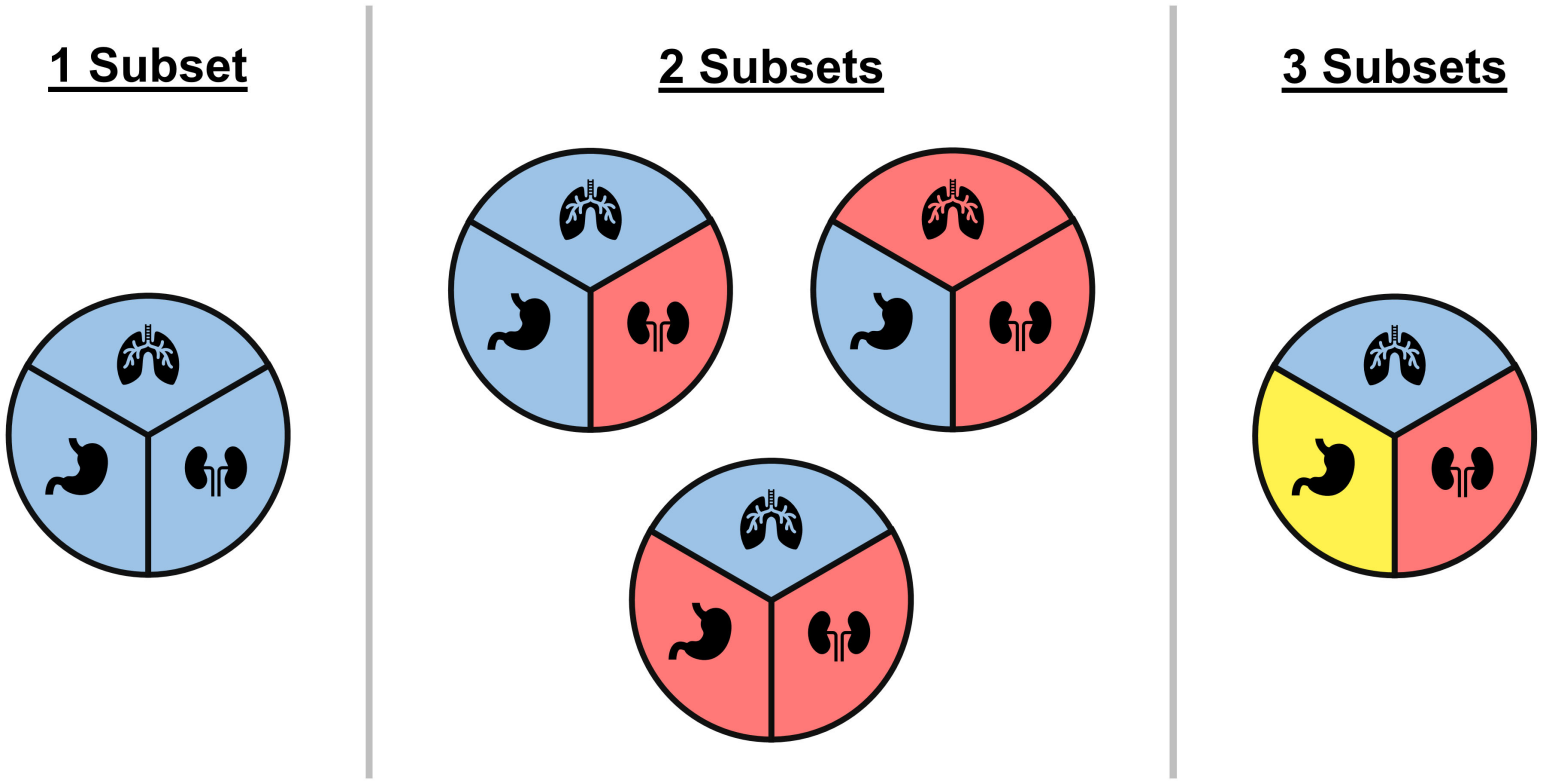
An illustrative example of all possible ways of classifying three tumor types into subgroups, where each circle represents a unique classification and colors correspond to different subgroups within a classification. Under the BMA approach of Psioda et al. (51), each classification corresponds to a unique model where tumor types within a subset are assumed to have equivalent response rates while differing from tumor types in other subsets. In the case of three tumor types, one model assumes all three tumor types have the same response rate, three models assume that two tumor types share a response rate that is distinct from the third tumor type, and one model assumes all three tumor types have distinct response rates.

The simplest of the model averaging methods assumes heterogeneity in rates can be captured with only two models (49): a model assuming response rates are all equivalent, and a model that groups tumor types as either active or inactive. An alternative approach is to group all tumor types into either an inactive group with low response rate or an active group with a high response rate (50). Psioda et al. (51) propose an approach that considers all possible classifications of tumor types into subsets, where tumor types within a subset are assumed to share a distinct response rate that differs from the response rates in other subsets (see **Figure 3**). The method does not force the subsets into arbitrary groups of active or inactive, but instead allows the data to dictate which tumor types are similar enough to borrow information from each other. Hobbs et al. (52) proposed a version that restricted model averaging to only pairwise combination of tumor types.

#### 3.2.4 Other design types and methods

Other design methods incorporate information borrowing by pooling all tumor types if deemed homogeneous at the interim analysis (53) or by pooling within subgroups that are defined using a clustering algorithm (54), clustering of tumor types (55–58), modeling patient-level data with a tree ensemble method (59), and using Bayesian commensurate priors (60). Baumann et al. (61) propose conditions that can improve the behavior of posterior probabilities when applied to various Bayesian approaches for basket trials. Further information on these methods, including their limitations and software availabilities, are listed in **Supplementary File 3**.

#### 3.2.5 Advantages and disadvantages of the methods

An advantage of most pruning-and-pooling methods lies in the simplicity of the design and analysis. This simplicity, however, brings several limitations. By pooling tumor types during the second stage, tumor types may be pruned prematurely based on limited data from each tumor type at the time of the interim analysis. Further, these methods implicitly assume that all tumor types can be categorized as either active or inactive, failing to allow for potential differences in the magnitudes of the tumor-specific response rates.

The conventional BHM discussed by Thall et al. (34) and Berry et al. (35) is motivated by the exchangeability assumption which may not be appropriate if, for example, a single tumor type has high activity while others have none. The amount of information borrowing is also sensitive to the choice of prior information on the between-tumor variance. Several of the proposed extensions to the BHM relax the exchangeability assumption, allowing one to better discern the extent to which information should be borrowed across tumor types at the cost of increased computational intensity.

A major strength of some model averaging methods, such as the approaches of Psioda et al. (51) and Hobbs et al. (52), is the flexibility to consider all possible structures of heterogeneity among tumor types. With the increased model flexibility, however, comes greater computational intensity.

### 3.3 Comparative analysis of basket trial methods

The previous section reviewed the proposed methods for basket trial design and analysis by highlighting their key features. Most methods were initially proposed by demonstrating better performance over one or two existing methods with respect to selected operating characteristics (e.g., increased statistical power, lower type I error rates), primarily through simulation studies. When using simulation studies to highlight the benefits of newly proposed methods, the relative performance of the methods can sometimes be exaggerated depending on the simulation setting. In this section, we illustrate the application of two different classes of information borrowing methods—Bayesian hierarchical modeling and model averaging—to data published from completed trials. To the best of our knowledge, there have been very few cases where the results of the methods were compared using actual data from a representative collection of basket trials.

For the comparative analysis of basket trial methods in this section, we compare and contrast the inferences based on the selected methods with respect to the probability that tumor-specific ORRs are greater than some threshold, and we note that it is not possible to assess the type I error rate, power, or other measures of statistical performance (e.g., gains in precision for estimates of ORR) by analyzing data from a specific trial where the ultimate truth is not known. With any clinical development plan, it is important to assess the risk of type I error rates and type II error rates (i.e., false negative rates)—both at the tumor and trial levels—using simulation studies that imitate clinical scenarios under both the null hypothesis (to assess type I error rates) and the alternative hypothesis (to assess power and type II error rates). The same is applicable to the design of basket trials. **Supplementary File 3** includes a list of which methods have been compared to one another via simulation studies in methodological papers, and we refer readers to these papers for a more comprehensive understanding of the operating characteristics of the different methods in a variety of scenarios.

We conducted analyses of data from 12 completed baskets trials with published ORRs per tumor type (**Supplementary File 4**). For each trial, we estimated the tumor-specific ORRs and the probability that each is greater than the response rate under the standard of care (SoC) or a pre-specified meaningful clinical threshold using three methods: a Bayesian model without information borrowing (IND), the calibrated Bayesian hierarchical model (CBHM) proposed by Chu et al. (44), and the Bayesian model averaging (BMA) approach of Psioda et al. (51). From the class of Bayesian hierarchical models, we selected the CBHM due to its demonstrated ability to control the tumor-specific type I error rate compared to other variations of the BHM (44), and we selected the BMA approach from the class of model averaging methods due to its demonstrated comparability with the CBHM with respect to statistical performance (51). While we use these two methods to represent their respective classes of information borrowing methods, we do not intend to advocate for one method being uniformly better than another. In fact, the authors believe no such method exists.

#### 3.3.1 Homogeneous basket trials

A common assumption in the analysis of basket trials is a similar response rate across tumor types. While this assumption is often not practical, a phase I/II study of hRS7-SN38 Antibody Drug Conjugate in patients with epithelial cancer (NCT01631552) serves as an example where homogeneity in estimated tumor-specific response rates was observed (**Table 2**). The tumor types had unequal sample size with the triple negative breast cancer (TNBC) cohort being the largest tumor type (108 participants) and the metastatic urothelial cancer (mUC) cohort being the smallest tumor type (45 participants). Both the BMA approach and the CBHM are expected to borrow information across tumor types to augment the information available in tumor types with smaller number of participants. The estimated ORR for metastatic urothelial cancer) (mUC) by the BMA approach and the CBHM borrowed information from the TNBC and the cohort with hormone receptor positive (HR+) or human epidermal growth factor receptor 2 negative (HER2-). In agreeance with the findings of Psioda et al. (51), both the BMA approach and the CBHM provided similar results, both in terms of response rates as well as the posterior probability in support of the efficacy of the treatment. We observe similar results with the IND approach due to the large sample sizes for each tumor type, however, we would expect the advantages of the information borrowing methods to become more apparent as the sample sizes decrease. In such a case, the results from the IND approach would be more susceptible to a greater degree of variation due to limited enrollment for any of the tumor types, whereas the CBHM and the BMA approach can estimate the tumor specific ORRs with greater precision through borrowing information across tumor types demonstrating similar treatment effects.

**Table 2:** Example of a basket trial with homogeneous response rate across tumor types (ClinicalTrials.gov ID: NCT01631552).

	Observed Data			Trial Criteria		Estimated ORR (Posterior Probability)		
Tumor Type/Basket	#Participants	#Responders	Response Rate	SoC ORR	Targeted ORR	IND	BMA	CBHM
TNBC	108	36	0.333	0.10*	0.30*	0.332 (1.000)	0.325 (1.000)	0.324 (1.000)
HR+/HER2- mBC	54	17	0.315	0.10*	0.30*	0.313 (1.000)	0.315 (1.000)	0.316 (1.000)
mUC	45	13	0.289	0.10*	0.30*	0.287 (1.000)	0.304 (1.000)	0.309 (1.000)

*Trial criteria values not reported in trial publications, values instead assumed by study authors for the comparative analysis.

Acronyms: TNBC (triple negative breast cancer), HR+ (hormone receptor positive), HER2- (human epidermal growth factor receptor 2 negative), mBC (metastatic breast cancer), mUC (metastatic urothelial cancer). Note that the posterior probability is P(ORR > ORR_SoC_|Data).

#### 3.3.2 Basket trials with non-zero response rate in only one tumor type

Analysis of a phase II trial of the cyclin-dependent kinase inhibitor in patients with cancer (NCT01037790), and a study to assess safety and efficacy of the second mitochondria-derived activator of caspases (SMAC) mimetic (NCT04122625) are presented in **Table 3**. The BMA approach shows consistent results with the analysis without information borrowing (IND), an indication that the BMA approach borrowed little or no information across the tumor types with non-zero response rates. However, the impact of a small number of participants can be seen in the results. The higher the number of participants per tumor type with no responder, the closer the estimated response rates were to zero. The CBHM treated the response rates as homogeneous, particularly in NCT04122625 where the tumor types have the same estimated response rates due to pooling of information to compensate for the small number of participants per tumor type. It also means that the variability around the overall response rate across tumor type was very small, an indication that the measure of homogeneity in the CBHM could not discriminate between the tumor types. All the methods provided weak evidence in support of treatment efficacy and are likely to result in the same conclusion.

**Table 3:** Examples of basket trial where all but one tumor types have zero response rates.

	Observed Data			Trial Criteria		Estimated ORR (Posterior Probability)		
Tumor Type/Basket	#Participants	#Responders	Response Rate	SoC ORR	Targeted ORR	IND	BMA	CBHM
ClinicalTrials.gov ID: NCT01037790								
Breast carcinoma	63	4	0.063	0.15	0.35*	0.066 (0.014)	0.063 (0.011)	0.060 (0.009)
Colorectal	18	0	0.000	0.15	0.35*	0.013 (0.005)	0.013 (0.003)	0.004 (0.001)
Esophageal and/or gastric	19	0	0.000	0.15	0.35*	0.013 (0.004)	0.012 (0.002)	0.004 (0.001)
CRU-GCT	30	0	0.000	0.15	0.35*	0.008 (0.001)	0.008 (0.000)	0.003 (0.000)
Other	11	0	0.000	0.15	0.35*	0.021 (0.021)	0.019 (0.012)	0.006 (0.003)
ClinicalTrials.gov ID: NCT04122625								
SCLC	8	0	0.000	0.05	0.15	0.011 (0.067)	0.013 (0.084)	0.029 (0.167)
H&N SCC	8	0	0.000	0.05	0.15	0.011 (0.067)	0.013 (0.084)	0.029 (0.167)
Gastrointestinal	8	0	0.000	0.05	0.15	0.011 (0.067)	0.013 (0.084)	0.029 (0.166)
Ovarian & endometrial	11	1	0.091	0.05	0.15	0.092 (0.618)	0.084 (0.576)	0.029 (0.169)

*Trial criteria values not reported in trial publications, values instead assumed by study authors for the comparative analysis.

Acronyms: CRU-GCT (cisplatin-refractory unresectable germ cell tumors), SCLC (small cell lung cancer), H&N SCC (squamous cell carcinoma of the head and neck). Note that the posterior probability is P(ORR>ORR_SoC_|Data).

#### 3.3.3 Basket trials with zero response rate in only one tumor type

**Table 4** presents the results of (1) a phase Ia/IIa trial investigating the safety, tolerability, and antitumor activity of a monoclonal antibody mixture targeting MET in patients with advanced solid tumor malignancies (NCT02648724); (2) a modular phase II study to link targeted therapy to patients with RAS/RAF/MEK activated tumors (NCT01885195); and (3) studies of temozolomide in combination with topotecan in refractory and relapsed paediatric solid tumors (NCT00918320). In NCT02648724, the results from the BMA approach and the CBHM approach were generally consistent with the results from the analysis without information borrowing. In this example there was no obvious impact of the information borrowing method because the homogeneous subset of NSCLC MET-amplified and NSCLC METEx14DEL had a similar response rate and comparable sample size. However, the BMA approach was more conservative than the other methods when estimating the response rates for multiple myeloma and acute myeloid leukaemia in NCT01885195 due to a big difference in the number of participants per tumor type. The results from the CBHM were not influenced by the huge difference in number of participants, which could be due to the fact that the measure of homogeneity in the CBHM is not affected by a small number of participants. NCT00918320 presents an interesting case where the response rates for the homogeneous subgroup were not the same. The BMA approach borrowed information from the Neuroblastoma tumor type, which had the largest number of participants, and it resulted in a smaller posterior probability in support of treatment efficacy for the central nervous system tumors as compared to the CBHM or analysis without information borrowing. These results illustrate that an information borrowing method can improve or decrease the level of confidence in support of treatment efficacy depending on the strength of the treatment effect and the number of participants per tumor type.

**Table 4:** Basket trials where only one tumor type has zero or close to zero response rate.

	Observed Data			Trial Criteria		Estimated ORR (Posterior Probability)		
Tumor Type/Basket	#Participants	#Responders	Response Rate	SoC ORR	Targeted ORR	IND	BMA	CBHM
ClinicalTrials.gov ID: NCT02648724								
Basket cohort	25	0	0.000	0.10*	0.30*	0.008 (0.006)	0.009 (0.009)	0.001 (0.000)
NSCLC MET-amplified cohort	8	2	0.250	0.10*	0.30*	0.244 (0.859)	0.244 (0.887)	0.252 (0.855)
NSCLC METEx14Del cohort	12	3	0.750	0.10*	0.30*	0.246 (0.915)	0.246 (0.928)	0.249 (0.908)
ClinicalTrials.gov ID: NCT01885195								
Solid tumors (non-hematologic)	104	3	0.029	0.10*	0.30*	0.030 (0.002)	0.031 (0.002)	0.029 (0.001)
Multiple myeloma	3	1	0.333	0.10*	0.30*	0.300 (0.815)	0.287 (0.788)	0.334 (0.814)
Acute myeloid leukemia	3	1	0.333	0.10*	0.30*	0.300 (0.815)	0.287 (0.788)	0.335 (0.812)
ClinicalTrials.gov ID: NCT00918320								
Neuroblastoma	38	7	0.184	0.20	0.40	0.187 (0.387)	0.185 (0.375)	0.183 (0.367)
CNS tumors	33	7	0.212	0.20*	0.40*	0.215 (0.552)	0.203 (0.483)	0.211 (0.529)
Other solid tumors	32	2	0.063	0.20*	0.40*	0.070 (0.012)	0.082 (0.023)	0.064 (0.009)

*Trial criteria values not reported in trial publications, values instead assumed by study authors for the comparative analysis.

Acronyms: NSCLC (non-small cell lung cancer), CNS (central nervous system). Note that the posterior probability is P(ORR > ORR_SoC_|Data).

#### 3.3.4 Basket trials with heterogeneous response rates

**Figure 4:**
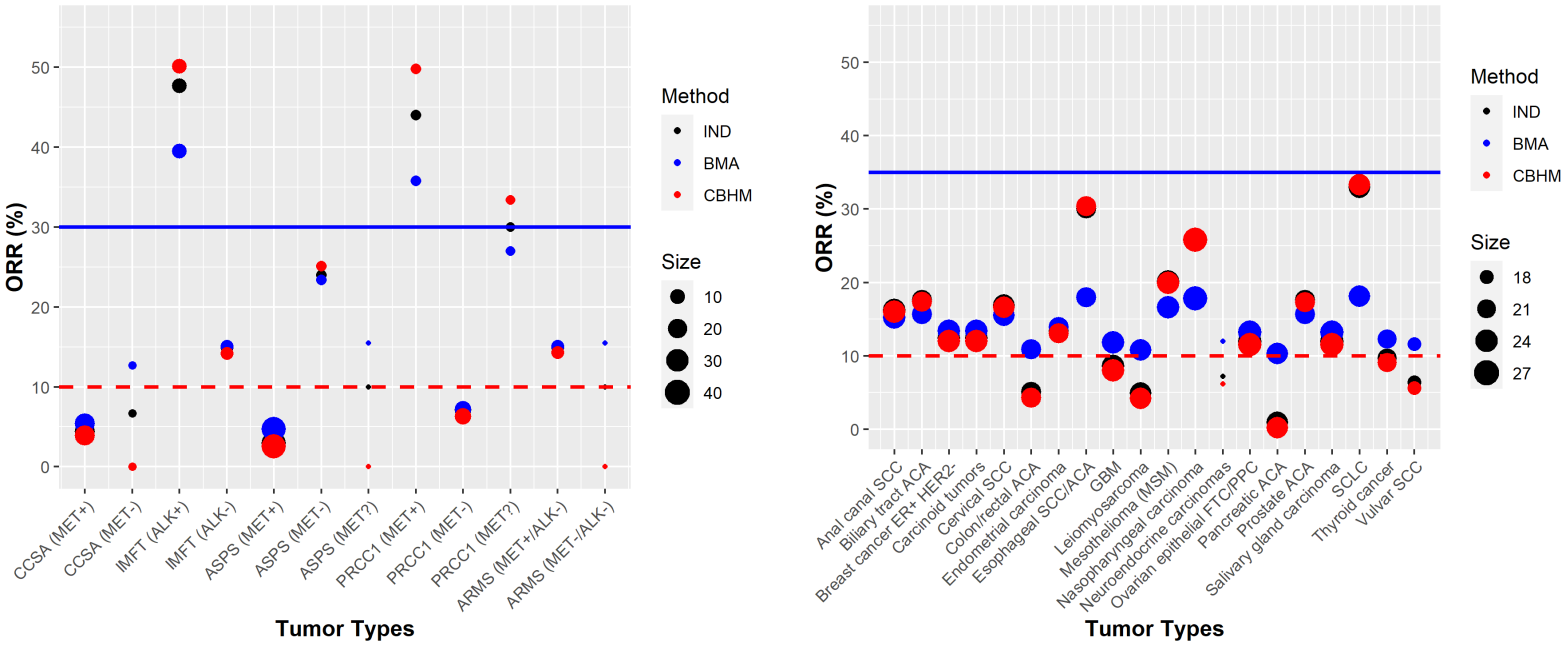
Examples of trials with heterogeneous response rates NCT01524926 (left panel) and NCT02054806 (right panel). The red dotted line denotes the response rate under standard of care or a clinical meaningful threshold. The solid blue line represents the targeted response rate to justify further development of the investigational product.

Most basket trials are likely to have heterogeneous response rates, and the degree of information borrowing across tumor types will depend on the level of heterogeneity that is anticipated and/or observed. **Figure 4** shows an example of a completed basket trial with heterogeneous response rates. This trial is a cross-tumoral phase II clinical trial exploring crizotinib in patients with advanced tumors induced by causal alterations of ALK and/or MET (NCT01524926). The trial had twelve baskets characterized by tumor types and ALK/MET alterations, each sharing a common threshold for the standard of care or a common clinical meaningful threshold. Furthermore, the number of participants differs substantially between the baskets. The results show no evidence in support of a tumor agnostic effect independent of which method was used, although the IMFT (ALK+) and PRCC1 (MET+) baskets achieved more than the targeted response rate of 30% (across all methods). The CBHM borrowed little or no information across the baskets due to the high level of heterogeneity between the response rates. The amount of information borrowed by the BMA approach depends on the homogeneous subsets. It is interesting to note that the estimated response rates for IMFT (ALK+) and PRCC1 (MET+) by the BMA approach were smaller than the estimated response rates by both the analysis without information borrowing and the CBHM due to the small number of participants in the baskets (see **Supplementary Table S5 in Supplementary File 1**).

Unlike NCT01524926, a phase Ib study of pembrolizumab in participants with select advanced solid tumors (NCT02054806) had heterogeneous response rates and a comparable sample size of around 20 participants per tumor type. The results from the CBHM approach aligned more closely with the results from the analysis without information borrowing, an indication that the CBHM borrowed little or no information due to the heterogeneous response rates. Compared to the CBHM, the BMA approach overestimated or underestimated the response rates depending on the subsets of homogeneous tumor types; e.g., the response rates for esophageal SCC/ACA, nasopharyngeal carcinoma, and small-cell lung cancer (SCLC) were estimated to be approximately 18% instead of the observed 30%, 26%, and 33%, respectively. The estimated response rates by the analysis without information borrowing were nested between the results from the BMA approach and the CBHM, hence, the results are not obvious from the plots in **Figure 4**. The posterior probabilities from all the methods in most cases would lead to a similar conclusion in support of treatment efficacy for all the tumor types. Additional results are presented in **Supplementary Table S5 of Supplementary File 1**.

### 3.4 Missed opportunity?

The current basket trial designs and practices focus on an individual phase of a CDP, particularly phase Ib or phase II. As a core part of the development of innovative statistical methodology for study design or data analysis, simulation studies are typically undertaken to compare the performance of the chosen method to other approaches with respect to some operating characteristic (e.g., chance of false efficacy conclusion or type I error at the tumor type and trial level). Simon (62) discusses the possibility of conducting an extended phase II or a phase III trial following a basket trial for tumor types which are not rare, however, this work includes no systematic investigation into the degree to which the inclusion of a basket trial in a CDP might add value.

To address the gap, we provide a brief illustration to demonstrate how incorporating a basket trial into a larger CDP might impact the success of the subsequent development program. For ease of exposition, we consider a simple, hypothetical CDP consisting of a single arm phase Ib basket trial with five tumor types (10 participants per tumor type) and subsequent randomized phase II trials for any tumor types in which signal is detected in the phase Ib basket trial. Each phase II trial assumed a total of 124 participants with equal allocation of 62 participants per arm (chosen to ensure at least 80% power to detect a risk difference of 20% in a phase II trial). Both the phase Ib and phase II trials measure objective response as the primary endpoint and investigate the same line of therapy with a minimum medicinal profile of 30% response rate against a response rate of 10% for the standard of care (a minimum medicinal profile is a company’s projection of the smallest benefit for an investigational product necessary to achieve both commercial and regulatory success).

We evaluated the impact of the basket trial on the subsequent phase II trials using simulation studies, and we consider six scenarios with respect to the underlying tumor-specific response rates in the phase Ib trial. Specifically, we vary the ratio of active tumor types to inactive tumor types (i.e., underlying ORRs of 30% and 10%, respectively), where the 5-active-tumors scenario is motivated by trial NCT01631552 and the heterogeneous scenarios are motivated by trial NCT01885195. For any tumor type in which signal is detected in phase Ib, the treatment arm of the subsequent phase II trial is assumed to have the same underlying tumor-specific ORR as defined in the phase Ib trial under the given scenario, whereas the control arm is assumed to have an ORR of 10% to reflect a SoC.

We simulated 100,000 hypothetical phase Ib and phase II trials under each scenario. For each phase Ib dataset, we applied both an independent (IND) Bayesian analysis for each tumor type without information borrowing and the BMA approach to compute the posterior probability that the response rate in a given tumor type was greater than the response rate under a SoC. For each approach, we declared the treatment to be successful (i.e., signal detected) for a given tumor type if the associated posterior probability exceeded 80%. In the phase II analysis, the two arms were compared using a standard Bayesian analysis to test the difference between two proportions, and the treatment was declared successful in phase II if the posterior probability of the treatment response rate being greater than the control response rate exceeded 90%. After repeating this process for each set of simulated studies, we calculated the phase Ib probability of success (PoS) for each tumor type as the percentage of simulated phase Ib trials in which the treatment was declared successful for the corresponding tumor type, and we calculated the joint PoS as the percentage of simulated studies in which the treatment was declared successful in both the phase Ib and phase II trials. Additional details of the simulation study design can be found in **Supplementary File 1**.

**Figure 5:**
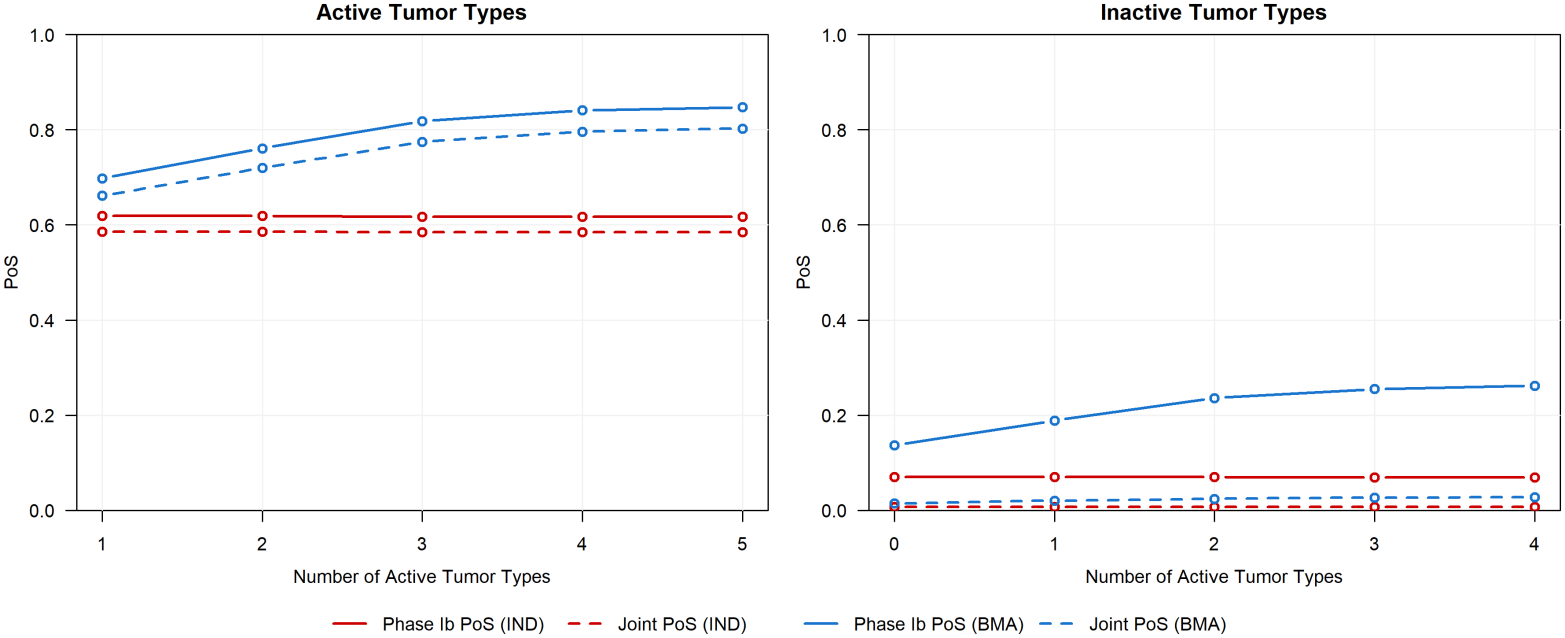
Phase Ib PoS and joint PoS for active tumor types (left panel, ORR of 30%) and inactive tumor types (right panel, ORR of 10%) across various scenarios that differ in the number of active tumor types.

**Figure 5** presents the simulation results. In each scenario, both the phase Ib PoS and joint PoS for active tumor types are greater when the BMA approach is used in phase Ib compared to when the IND approach is used. As the number of active tumor types increases, both measures of PoS under the IND approach remain constant, whereas these probabilities increase under the BMA approach due to information borrowing across additional active tumor types, ultimately resulting in a higher probability that an active tumor type successfully continues past both the phase Ib and phase II trials. The increase in information borrowing also results in an increased type I error rate for inactive tumor types, however, the joint PoS is only marginally greater when the BMA approach is used in phase Ib compared to the IND approach. While information borrowing approaches moderately increase the probability that an inactive tumor type continues into phase II, the probability of it progressing into a more expensive phase III trial remains low. Further, these approaches can also improve precision when estimating tumor-specific ORRs (see **Supplementary File 1** for results). By evaluating the impact of a basket trial on multiple phases of a CDP rather than focusing on only the operating characteristics of the basket trial alone, sponsors can make more informed decisions relating to the progression of tumor types into later expensive phase III trials by understanding the benefits and risks of the basket trial design and the modeling approach under different scenarios.

**Figure 6:**
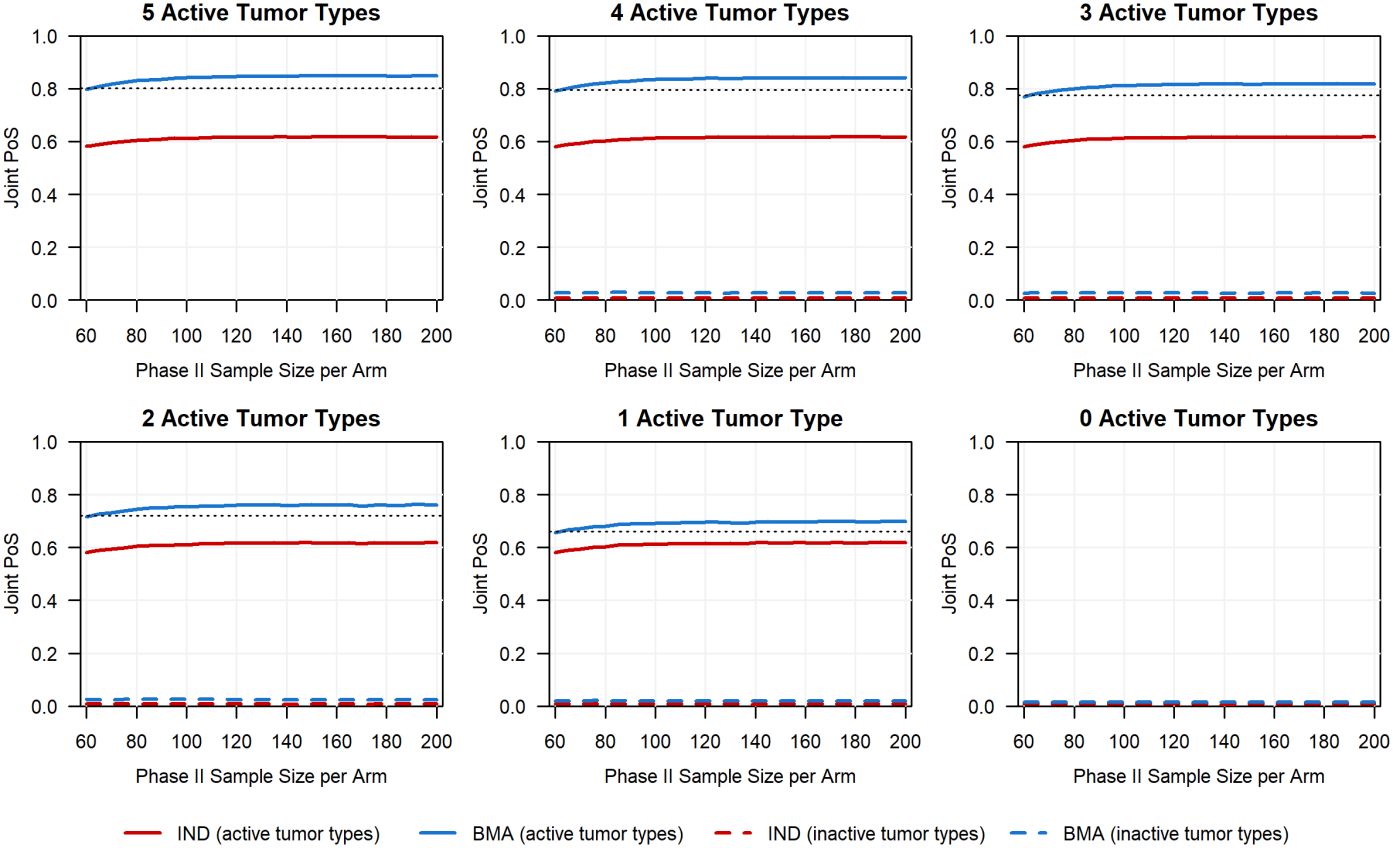
Planned phase II sample size per arm versus the joint PoS for all six scenarios in which we range the number of active tumor types (ORR of 30%) between 0 and 5. The black horizontal dashed line for the first five scenarios corresponds to the joint PoS obtained for active baskets if using the BMA approach in phase Ib and a planned phase II sample size of 62 per arm (i.e., sample size chosen to ensure at least 80% power to detect a risk difference of 20% in a phase II trial).

To further highlight the advantages of assessing multiple phases within a CDP, we illustrate how the choice of modeling approach in phase Ib and the cost of the CDP (measured by phase II sample size) influence the joint PoS, as shown in **Figure 6**. In each scenario, the joint PoS under the IND approach quickly plateaus despite the increase in the planned phase II sample size per arm, remaining well below the joint PoS obtained using the BMA approach in phase Ib and a phase II sample size of 62 participants per arm (denoted in **Figure 6** by a horizontal dashed line). While the observed differences in joint PoS are primarily driven by the phase Ib PoS under each approach, these results show that further investment in phase II following the use of the IND approach in phase Ib will not make up the discrepancy in joint PoS of the two basket trial modeling approaches for these specific scenarios. If we were to instead increase the sample size of each tumor type in phase Ib (e.g., 20 per tumor type), the joint PoS trajectory under the IND approach may eventually reach the joint PoS that was obtained with the BMA approach and 62 participants per arm in phase II, although a much larger phase II sample size may be required following the use of IND approach to obtain an equivalent joint PoS (see additional simulation results in **Supplementary File 1** for the case when phase Ib sample sizes are set as 20 for each tumor type). This highlights that the impact of the basket trial design with information borrowing methods does not stop at the end of phase Ib but has a cascade effect that impacts the joint probability of success and cost in phase II. This wholistic view of the clinical development plan and the impact of a decision made in an early phase on later expensive clinical development phases motivates the need for information borrowing and basket trial design in early phase oncology where most companies rarely invest in a moderate to large number of participants.

## 4 Discussion

Our systematic review of basket trials in practice shows increasing usage of basket trial designs in oncology, which is consistent with the trend reported by other recent reviews (11–13). The current practice is largely in the design of single-arm phase II trials, although basket trials are also becoming more common in the design of phase Ib trials. Increasing adoption of basket trial design creates a positive shift in phase Ib and phase II trials which historically involve a small number of participants. Most basket trials in practice use ORR or another binary endpoint, and few studies use innovative information borrowing methods. This represents a significant gap in basket trial design in oncology given the lack of sensitivity of ORR and its potential disconnect from overall survival (63). An important area for research in basket trial design and analysis is the extension of innovative information borrowing methods beyond single-arm phase Ib and phase II trials, including the development of methods that address the challenges associated with longer-term endpoints such as large number of participants, data maturity, treatment switching, and other intercurrent events.

Our review of basket trials in practice and proposed statistical methodology has limitations common to systematic reviews. When searching for abstracts relevant to basket trials, only the abstracts that met our defined inclusion criteria for the search were deemed as eligible, potentially resulting in some abstracts not being identified through our search. For example, abstracts that discuss the results of a basket trial without using the phrase “basket trial” or “basket clinical trial” were not returned in the search, and hence the number of basket trials identified in our review is likely underestimated. Additionally, trials and methodological papers were only considered if they met the respective inclusion criteria for each review as defined by the team. While these criteria were defined to allow for objective decision making relating to the inclusion of trials and methods, we note that these criteria may differ from the criteria used in other systematic reviews for either the practice of basket trials or the statistical methodology. Our inclusion of studies that investigated only one treatment regimen across multiple tumor types or biomarker defined subgroups within the same tumor means that studies with a combined feature of basket and umbrella trials were excluded from our review. We also noticed inconsistencies in the reporting of basket trial results, specifically with investigators publishing the results for the most promising tumor types. Results for all tumor types in basket trials should be published directly on ClinicalTrials.gov to reduce reporting bias and increase transparency of trial results.

Several information borrowing methods assume that tumor types in a basket trial design will readout around the same time and data from all tumor types will be available for pooled analysis or information borrowing both at the interim and final analysis. However, this may not always be the case due to factors such as differences in the prevalence of each tumor type or varying recruitment rates. Although tumor types in a basket trial are not required to have the same number of participants, it is important to assess the feasibility of recruitment at the design stage. Where basket trial design with information borrowing may delay the clinical development plan, the cost of the delay—both for patients and the company—may outweigh the benefit of information borrowing methods. Note that the BMA approach is adaptable, allowing sponsors to place constraints on a tumor-specific minimum number of participants and to determine at each interim look whether enough data have been collected from a tumor type to examine for futility or efficacy.

A comparative analysis of basket trial methods using data from the completed trials indicates that neither complete homogeneous nor complete heterogeneous response rates are practical assumptions for the design of a basket trial in oncology. We recommend the use of innovative information borrowing methods for both the design and analysis of a basket trial to minimize the risk of false positive or false negative conclusion, particularly when small and/or unequal number of participants is envisaged. We also observed a disconnect between the biological expectation of a tumor agonistic effect and the observed heterogeneous response rates between tumor types with a common molecular alteration or biomarker. Most of the current methods focus on only a single endpoint with no adjustment for covariates to reduce the heterogeneity in the outcome data. This approach ignores the different sources of variability, which include tumor-to-tumor heterogeneity, tumor-site interactions, and clinical heterogeneity between patients. Future research which adjusts for known sources of variability using non-targeted biomarkers and other prognostic factors may help to explain what currently appears as heterogeneity between tumor types in basket trials. Our approach to empirical comparison of the methods is specific to the setting of each trial. In practice, innovative methods should be tailored to each trial. Simulation methods provide a flexible scheme to compare performance of different methods, and existing papers on basket trial methods often report a simulation study comparing their method with other selected methods.

Moreover, the current practice of basket trial design, which does not consider its impact on the CDP, may lead to a missed opportunity in improving the probability of success of a future trial. As highlighted by our simulation study, gating phase II with a phase Ib basket trial can reduce the size of phase II trials, and losses in the joint PoS for phase Ib and phase II as a result of not using an advanced inference method in phase Ib may not be recoverable by running a larger phase II trial. Simulation studies should be used to investigate the impact of basket trial design and other study design methods in an earlier clinical phase on the future confirmatory phase III trials. Among other things, future research should quantify the degree to which a basket trial de-risks a future trial and how this degree of de-risking differs when using information borrowing methods compared to independent analyses. It is also important to extend the current framework of basket trials to CDPs in which the basket trial and future confirmatory trials have different endpoints (e.g., binary versus time-to-event).

## Data availability statement

The original contributions presented in the study are included in the article/**Supplementary Material**. Further inquiries can be directed to the corresponding author.

## Author contributions

AK: Data curation, Investigation, Methodology, Project administration, Supervision, Validation, Visualization, Writing – original draft, Writing – review & editing. NB: Data curation, Formal Analysis, Investigation, Methodology, Validation, Visualization, Writing – original draft, Writing – review & editing. SH: Data curation, Investigation, Validation, Visualization, Writing – review & editing. T-TC: Conceptualization, Funding acquisition, Methodology, Resources, Writing – review & editing. HZ: Conceptualization, Funding acquisition, Investigation, Methodology, Resources, Writing – review & editing. MP: Conceptualization, Funding acquisition, Investigation, Methodology, Resources, Software, Visualization, Writing – review & editing.

## References

[1] La Thangue NB Kerr DJ . Predictive biomarkers: a paradigm shift towards personalized cancer medicine. Nat Rev Clin Oncol (2011) 8:587–96. doi: 10.1038/nrclinonc.2011.121 21862978

[2] Tateo V Marchese PV Mollica V Massari F Kurzrock R Adashek JJ . Agnostic approvals in oncology: getting the right drug to the right patient with the right genomics. Pharm (Basel Switzerland) (2023) 16:614. doi: 10.3390/ph16040614 PMC10144220 37111371

[3] FDA D.I.S.C.O . Burst Edition: FDA approvals of Retevmo (selpercatinib) for adult patients with locally advanced or metastatic RET fusion-positive solid tumors, and Retevmo (selpercatinib) for adult patients with locally advanced or metastatic RET fusion-positive non-small cell lung cancer (Accessed June 27, 2023).

[4] FDA grants accelerated approval to dabrafenib in combination with trametinib for unresectable or metastatic solid tumors with BRAF V600E mutation. Available at: https://www.fda.gov/drugs/resources-information-approved-drugs/fda-grants-accelerated-approval-dabrafenib-combination-trametinib-unresectable-or-metastatic-solid (Accessed June 5 2023).

[5] FDA D.I.S.C.O . Burst Edition: FDA approvals of Jemperli (dostarlimab-gxly) for patients with mismatch repair deficient recurrent or advanced solid tumors, and Opdivo (nivolumab) for the adjuvant treatment of patients with urothelial carcinoma. Available at: https://www.fda.gov/drugs/resources-information-approved-drugs/fda-disco-burst-edition-fda-approvals-jemperli-dostarlimab-gxly-patients-mismatch-repair-deficient (Accessed June 5, 2023).

[6] FDA approves entrectinib for NTRK solid tumors and ROS-1 NSCLC. Available at: https://www.fda.gov/drugs/resources-information-approved-drugs/fda-approves-entrectinib-ntrk-solid-tumors-and-ros-1-nsclc (Accessed June 27, 2023).

[7] FDA grants accelerated approval to pembrolizumab for first tissue/site agnostic indication . Available at: https://www.fda.gov/drugs/resources-information-approved-drugs/fda-grants-accelerated-approval-pembrolizumab-first-tissuesite-agnostic-indication (Accessed June 27, 2023).

[8] FDA approves larotrectinib for solid tumors with NTRK gene fusions . Available at: https://www.fda.gov/drugs/fda-approves-larotrectinib-solid-tumors-ntrk-gene-fusions (Accessed June 27, 2023).

[9] Woodcock J LaVange LM . Master protocols to study multiple therapies, multiple diseases, or both. New Engl J Med (2017) 377:62–70. doi: 10.1056/nejmra1510062 28679092

[10] Hirakawa A Asano J Sato H Teramukai S . Master protocol trials in oncology: Review and new trial designs. Contemp Clin Trials Commun (2018) 12:1–8. doi: 10.1016/j.conctc.2018.08.009 30182068 PMC6120722

[11] Park JJH Siden E Zoratti MJ Dron L Harari O Singer J . Systematic review of basket trials, umbrella trials, and platform trials: a landscape analysis of master protocols. Trials (2019) 20:572. doi: 10.1186/s13063-019-3664-1 31533793 PMC6751792

[12] Haslam A Olivier T Tuia J Prasad V . Umbrella review of basket trials testing a drug in tumors with actionable genetic biomarkers. BMC Cancer (2023) 23:46. doi: 10.1186/s12885-022-10421-w 36639625 PMC9840247

[13] Hobbs BP Pestana RC Zabor EC Kaizer AM Hong DS . Basket trials: review of current practice and innovations for future trials. J Clin Oncol (2022) 40:3520–8. doi: 10.1200/jco.21.02285 PMC10476732 35537102

[14] Moher D Liberati A Tetzlaff J Altman DG . Preferred reporting items for systematic reviews and meta-analyses: the PRISMA statement. BMJ (2009) 339:b2535–5. doi: 10.1136/bmj.b2535 PMC2714657 19622551

[15] Siden EG Park JJH Zoratti MJ Dron L Harari O Thorlund K . Reporting of master protocols towards a standardized approach: A systematic review. Contemp Clin Trials Commun (2019) 15:100406. doi: 10.1016/j.conctc.2019.100406 31334382 PMC6616543

[16] Park JJH Hsu G Siden EG Thorlund K Mills EJ . An overview of precision oncology basket and umbrella trials for clinicians. CA: A Cancer J Clin (2020) 70:125–37. doi: 10.3322/caac.21600 PMC7187272 32031692

[17] Meyer EL Mesenbrink P Dunger-Baldauf C Fülle H-J Glimm E Li Y . The evolution of master protocol clinical trial designs: A systematic literature review. Clin Ther (2020) 42:1330–60. doi: 10.1016/j.clinthera.2020.05.010 32622783

[18] Haslam A Olivier T Tuia J Prasad V . A systematic review of basket and umbrella trials in oncology: the importance of tissue of origin and molecular target. Eur J Cancer (2022) 178:227–33. doi: 10.1016/j.ejca.2022.10.027 36493558

[19] Higgins JPT Thomas J Chandler J Cumpston M Li T Page MJ . Cochrane handbook for systematic reviews of interventions version 6.4. Cochrane. (2023). Available at: www.training.cochrane.org/handbook.

[20] Eisenhauer EA Therasse P Bogaerts J Schwartz LH Sargent D Ford R . New response evaluation criteria in solid tumors: Revised RECIST guideline (version 1.1). Eur J Cancer (2009) 45:228–47. doi: 10.1016/j.ejca.2008.10.026 19097774

[21] Yin J Shen S Shi Q . Challenges, opportunities, and innovative statistical designs for precision oncology trials. Ann Trans Med (2022) 10:1038–8. doi: 10.21037/atm-22-356 PMC9577796 36267789

[22] Health C for D and R . Guidance for the use of Bayesian statistics in medical device clinical trials (2020). US Food and Drug Administration (Accessed June 21, 2023).

[23] Simon R . Optimal two-stage designs for phase II clinical trials. Controlled Clin Trials (1989) 10:1–10. doi: 10.1016/0197-2456(89)90015-9 2702835

[24] Palmer AC Plana D Sorger PK . Comparing the efficacy of cancer therapies between subgroups in basket trials. Cell Syst (2020) 11:449–460.e2. doi: 10.1016/j.cels.2020.09.003 33220857 PMC8022348

[25] Beckman R Antonijevic Z Kalamegham R Chen C . Adaptive design for a confirmatory basket trial in multiple tumor types based on a putative predictive biomarker. Clin Pharmacol Ther (2016) 100:617–25. doi: 10.1002/cpt.446 27509351

[26] Yuan SS Chen A He L Chen C Gause CK Beckman RA . On group sequential enrichment design for basket trial. Stat Biopharm Res (2016) 8:293–306. doi: 10.1080/19466315.2016.1200999

[27] Li W Zhao J Li X Chen C Beckman RA . Multi-stage enrichment and basket trial designs with population selection. Stat Med (2019) 38:5470–85. doi: 10.1002/sim.8371 31621949

[28] Chen C Li X Yuan S Antonijevic Z Kalamegham R Beckman RA . Statistical design and considerations of a phase 3 basket trial for simultaneous investigation of multiple tumor types in one study. Stat Biopharmaceutical Res (2016) 8:248–57. doi: 10.1080/19466315.2016.1193044

[29] Zhou H Liu F Wu C Rubin EH Giranda VL Chen C . Optimal two-stage designs for exploratory basket trials. Contemp Clin Trials (2019) 85:105807. doi: 10.1016/j.cct.2019.06.021 31260789

[30] Wu C Liu F Zhou H Wu X Chen C . Optimal one-stage design and analysis for efficacy expansion in Phase I oncology trials. Clin Trials (2021) 18:673–80. doi: 10.1177/17407745211052486 34693772

[31] Wu X Wu C Liu F Zhou H Chen C . A generalized framework of optimal two-stage designs for exploratory basket trials. Stat Biopharmaceutical Res (2021) 13:286–94. doi: 10.1080/19466315.2021.1906741

[32] Jing N Liu F Wu C Zhou H Chen C . An optimal two-stage exploratory basket trial design with aggregated futility analysis. Contemp Clin Trials (2022) 116:106741. doi: 10.1016/j.cct.2022.106741 35358718

[33] He L Ren Y Chen H Guinn D Parashar D Chen C . Efficiency of a randomized confirmatory basket trial design constrained to control the family wise error rate by indication. Stat Methods Med Res (2022) 31:1207–23. doi: 10.1177/09622802221091901 35404188

[34] Thall PF Wathen JK Bekele BN Champlin RE Baker LH Benjamin RS . Hierarchical Bayesian approaches to phase II trials in diseases with multiple subtypes. Stat Med (2003) 22:763–80. doi: 10.1002/sim.1399 12587104

[35] Berry SM Broglio KR Groshen S Berry DA . Bayesian hierarchical modelling of patient subpopulations: Efficient designs of Phase II oncology clinical trials. Clin Trials (2013) 10:720–34. doi: 10.1177/1740774513497539 PMC4319656 23983156

[36] Liu R Liu Z Ghadessi M Vonk R . Increasing the efficiency of oncology basket trials using a Bayesian approach. Contemp Clin Trials (2017) 63:67–72. doi: 10.1016/j.cct.2017.06.009 28629993

[37] Neuenschwander B Wandel S Roychoudhury S Bailey S . Robust exchangeability designs for early phase clinical trials with multiple strata. Pharm Stat (2015) 15:123–34. doi: 10.1002/pst.1730 26685103

[38] Chen C Hsiao C . Bayesian hierarchical models for adaptive basket trial designs. Pharm Stat (2023) 22:531–46. doi: 10.1002/pst.2289 36625301

[39] Chu Y Yuan Y . BLAST: bayesian latent subgroup design for basket trials accounting for patient heterogeneity. J R Stat Soc Ser C: Appl Stat (2018) 67:723–40. doi: 10.1111/rssc.12255

[40] Chen N Lee JJ . Bayesian cluster hierarchical model for subgroup borrowing in the design and analysis of basket trials with binary endpoints. Stat Methods Med Res (2020) 29:2717–32. doi: 10.1177/0962280220910186 32178585

[41] Jiang L Li R Yan F Yap TA Yuan Y . Shotgun: A Bayesian seamless phase I-II design to accelerate the development of targeted therapies and immunotherapy. Contemp Clin Trials (2021) 104:106338. doi: 10.1016/j.cct.2021.106338 33711459 PMC8180491

[42] Jiang L Nie L Yan F Yuan Y . Optimal Bayesian hierarchical model to accelerate the development of tissue-agnostic drugs and basket trials. Contemp Clin Trials (2021) 107:106460. doi: 10.1016/j.cct.2021.106460 34098036

[43] Takeda K Liu S Rong A . Constrained hierarchical Bayesian model for latent subgroups in basket trials with two classifiers. Stat Med (2021) 41:298–309. doi: 10.1002/sim.9237 34697822

[44] Chu Y Yuan Y . A Bayesian basket trial design using a calibrated Bayesian hierarchical model. Clin Trials (2018) 15:149–58. doi: 10.1177/1740774518755122 PMC5891374 29499621

[45] Lin R Thall PF Yuan Y . A phase I–II basket trial design to optimize dose-schedule regimes based on delayed outcomes. Bayesian Anal (2020) 16:179–202. doi: 10.1214/20-ba1205 PMC8277108 34267857

[46] Yin G Yang Z Odani M Fukimbara S . Bayesian hierarchical modeling and biomarker cutoff identification in basket trials. Stat Biopharm Res (2020) 13:248–58. doi: 10.1080/19466315.2020.1811146

[47] Liu S Takeda K Rong A . An adaptive biomarker basket design in phase II oncology trials. Pharm Stat (2022) 22:128–42. doi: 10.1002/pst.2264 36163614

[48] Jin J Riviere M Luo X Dong Y . Bayesian methods for the analysis of early-phase oncology basket trials with information borrowing across cancer types. Stat Med (2020) 39:3459–75. doi: 10.1002/sim.8675 32717103

[49] Asano J Hirakawa A . A Bayesian basket trial design accounting for uncertainties of homogeneity and heterogeneity of treatment effect among subpopulations. Pharm Stat (2020) 19:975–1000. doi: 10.1002/pst.2049 32779393

[50] Simon R Geyer S Subramanian J Roychowdhury S . The Bayesian basket design for genomic variant-driven phase II trials. Semin Oncol (2016) 43:13–8. doi: 10.1053/j.seminoncol.2016.01.002 26970120

[51] Psioda MA Xu J Jiang Q Ke C Yang Z Ibrahim JG . Bayesian adaptive basket trial design using model averaging. Biostatistics (2021) 22:19–34. doi: 10.1093/biostatistics/kxz014 31107534 PMC7846150

[52] Hobbs BP Landin R . Bayesian basket trial design with exchangeability monitoring. Stat Med (2018) 37:3557–72. doi: 10.1002/sim.7893 29984488

[53] Cunanan KM Iasonos A Shen R Hyman DM Riely GJ Gönen M . Specifying the true- and false-positive rates in basket trials. JCO Precis Oncol (2017) 1. doi: 10.1200/po.17.00181 PMC7446381 32913969

[54] Krajewska M Rauch G . A new basket trial design based on clustering of homogeneous subpopulations. J Biopharmaceutical Stat (2021) 31:425–47. doi: 10.1080/10543406.2021.1897993 34236938

[55] Fujikawa K Teramukai S Yokota I Daimon T . A Bayesian basket trial design that borrows information across strata based on the similarity between the posterior distributions of the response probability. Biometrical J Biometrische Z (2020) 62:330–8. doi: 10.1002/bimj.201800404 31608505

[56] Zhou T Ji Y . RoBoT: a robust Bayesian hypothesis testing method for basket trials. Biostatistics (2021) 22:897–912. doi: 10.1093/biostatistics/kxaa005 32061093

[57] Belay SY Guo X Lin X Xia F Xu J . Bayesian basket trial design accounting for multiple cutoffs of an ambiguous biomarker. Stat Biopharmaceutical Res (2022) 14:342–8. doi: 10.1080/19466315.2022.2029555

[58] Liu Y Kane M Esserman D Blaha O Zelterman D Wei W . Bayesian local exchangeability design for phase II basket trials. Stat Med (2022) 41:4367–84. doi: 10.1002/sim.9514 PMC10279458 35777367

[59] Pan J Bunn V Hupf B Lin J . Bayesian Additive Regression Trees (BART) with covariate adjusted borrowing in subgroup analyses. J Biopharmaceutical Stat (2022) 32:613–26. doi: 10.1080/10543406.2022.2089160 35737650

[60] Zheng H Wason JMS . Borrowing of information across patient subgroups in a basket trial based on distributional discrepancy. Biostatistics (2022) 23:120–35. doi: 10.1093/biostatistics/kxaa019 PMC8759447 32380518

[61] Baumann L Krisam J Kieser M . Monotonicity conditions for avoiding counterintuitive decisions in basket trials. Biometrical J Biometrische Z (2022) 64:934–47. doi: 10.1002/bimj.202100287 35692061

[62] Simon R . New designs for basket clinical trials in oncology. J Biopharmaceutical Stat (2017) 28:245–55. doi: 10.1080/10543406.2017.1372779 28877003

[63] Hu C Wang M Wu C Zhou H Chen C Diede S . Comparison of duration of response vs conventional response rates and progression-free survival as efficacy end points in simulated immuno-oncology clinical trials. JAMA Network Open (2021) 4:e218175. doi: 10.1001/jamanetworkopen.2021.8175 34047794 PMC8164100

