# Supplementary material for: Basket trials in oncology: a systematic review of practices and methods, comparative analysis of innovative methods, and an appraisal of a missed opportunity
Source: Front Oncol. 2023 Nov 14;13:1266286. doi: 10.3389/fonc.2023.1266286 (PMC10684308; doi:10.3389/fonc.2023.1266286)
Supplement: Supplementary file 1 [file DataSheet_1.docx]

**B****asket trials in oncology: a systematic review of practices and methods, comparative analysis of innovative methods, and an appraisal of a missed opportunity**

Adetayo Kasim^1^*, Nathan Bean^2^, Sarah Jo Hendriksen^1^, Tai-Tsang Chen^3^, Helen Zhou^3^, and Matt Psioda^2^

^1^Disease Area Strategy, Oncology Biostatistics, GSK, United Kingdom

^2^Statistics and Data Science – Innovation Hub, GSK, Collegeville, PA 19426, United States

^3^Disease Area Strategy, Oncology Biostatistics, GSK, Collegeville, PA 19426, United States

*** Correspondence:**Corresponding Author
[adetayo.s.kasim@gsk.com](mailto:adetayo.s.kasim@gsk.com)

**SUPPLEMENTARY FILE 1**

Table of Contents

[1. Systematic Review of Basket Trial Practices 2](#_Toc911959296)

[1.1 Data sources and searches 3](#_Toc683659451)

[1.2 Study inclusion and exclusion criteria 4](#_Toc1020038557)

[1.3 Data extraction 4](#_Toc444784871)

[1.4 Data synthesis 4](#_Toc1566549985)

[2. Review of Basket Trial Methods 4](#_Toc572267371)

[2.1 Data sources and searches 5](#_Toc1956268278)

[2.2 Study inclusion and exclusion criteria 5](#_Toc692098656)

[2.3 Data extraction 5](#_Toc524435598)

[2.4 Data synthesis 5](#_Toc1858296737)

[3. EQUATOR Checklist 5](#_Toc361631239)

[4. PRISMA Diagrams 9](#_Toc1781537390)

[4.1 PRISMA diagram for the systematic review of basket trial practices 10](#_Toc785943116)

[4.2 PRISMA diagram for the systematic review of basket trial methods 11](#_Toc835575298)

[5. Additional Results from the Systematic Review of Basket Trial Practices 11](#_Toc636376766)

[5.1 Number of patients 12](#_Toc2140384427)

[5.2 Number of tumor types, biomarkers, and prior lines of therapy 12](#_Toc404032845)

[5.3 Design and analytical methods 12](#_Toc342934491)

[6. Comparative Analysis of Completed Trials 13](#_Toc1956054638)

[6.1 Analysis methods 14](#_Toc1249885374)

[6.1.1 Notation 14](#_Toc356259901)

[6.1.2 Independent analyses within each tumor (i.e., no information borrowing) 14](#_Toc66652416)

[6.1.3 Bayesian model averaging 14](#_Toc1514590466)

[6.1.4 Calibrated Bayesian hierarchical model 15](#_Toc1868250634)

[6.2 Results for trials with heterogeneous response rates 15](#_Toc1390593339)

[7. Simulation Setting: Technical Information 18](#_Toc1063899020)

[7.1 Study design characteristics 19](#_Toc1618783252)

[7.2 Simulation parameters 19](#_Toc1278027386)

[7.3 Statistical analysis methods for phase Ib trial 20](#_Toc1663549096)

[7.4 Posterior probabilities and success criteria 20](#_Toc1837023423)

[7.5 General simulation setup 20](#_Toc1448698970)

[8. Additional Simulation Results 22](#_Toc1581345772)

[8.1 Simulation results with a phase Ib sample size of 10 per tumor type 23](#_Toc1663335175)

[8.2 Simulation results with a phase Ib sample size of 20 per tumor type 23](#_Toc397123799)

[9. Systematic Review Protocol 25](#_Toc309342245)

[9.1 Introduction 26](#_Toc1653721413)

[9.2 Methods and analysis 28](#_Toc165425347)

[9.2.1 External search strategy: basket trial practice 28](#_Toc930802214)

[9.2.2 External search strategy: statistical methods 29](#_Toc94161457)

[9.2.3 Internal GSK search strategy 30](#_Toc1872067922)

[9.2.4 Study inclusion and exclusion criteria 30](#_Toc256653982)

[9.2.5 Data extraction 31](#_Toc866121957)

[9.2.6 Risk of bias 31](#_Toc1606902104)

[9.2.7 Data synthesis and analysis 32](#_Toc697817275)

[9.3 Changes made to the systematic review protocol 32](#_Toc1394307039)

[10. References 33](#_Toc1038900468)

# 1. Systematic Review of Basket Trial Practices

## 1.1 Data sources and searches

Systematic searches were conducted on February 20, 2023, in MEDLINE, Embase, and the Cochrane Central Register of Controlled Trials. The search strategy mirrored the approach of Park *et al*. [1], with minor modifications, such as modifying search terms to focus only on basket trials, and we supplemented the search with a review of bibliographies from included publications and trial registries (ClinicalTrials.gov) for registered basket trial protocols. Further details on the number of hits from each database are presented in Table S1 – S3.

**Table S1: Number of hits in Embase**

| **No.**​ | **Terms**​ | **Hits (as of 20 Feb 2023)**​ |
| --- | --- | --- |
| 1​ | “Basket trial” OR “basket clinical trial”​ | 435​ |
| 2​ | Oncology or cancer​ | 5,450,905​ |
| 3​ | Human​ | 26,910,543​ |
| 4​ | 1 and 2​ | 391​ |
| 5​ | 3 and 4​ | 375​ |
| 6​ | 5 Limited to English​ | 372​ |
| 7 ​ | 6 Limited to 2019 - 2023​ | 272 |

**Table S2: Number of hits in PubMed/MEDLINE**

| **No.**​ | **Terms**​ | **Hits (as of 20 Feb 2023)**​ |
| --- | --- | --- |
| 1​ | “Basket trial*”[tw] OR “basket clinical trial*”[tw]​ | 233​ |
| 2​ | Oncology or cancer​ | 4,491,654​ |
| 3​ | Human​ | 21,528,245​ |
| 4​ | 1 and 2​ | 215​ |
| 5​ | 3 and 4​ | 213​ |
| 6​ | 5 Limited to 2019 - 2023​ | 149​ |

**Table S3: Number of hits in Cochrane Central Register of Controlled Trials**

| **No.**​ | **Terms**​ | **Hits (as of 20 Feb 2023)**​ |
| --- | --- | --- |
| 1​ | “Basket trial” OR “basket clinical trial”​ | 70​ |
| 2​ | Oncology or cancer​ | 1109​ |
| 3​ | Human​ | 8,858​ |
| 4​ | 1 and 2​ | 48​ |
| 5​ | 3 and 4​ | 46​ |
| 6​ | 5 Limited to 2019 – 2023​ | 29​ |

## 1.2 Study inclusion and exclusion criteria

To build upon the systematic review of basket trials by Park *et al*. [1], we considered only abstracts and papers published from January 2019 through February 2023 as eligible for the review of basket trial practices. In addition to the trials already identified by Park *et al*. [1], we searched for any unique basket trials referenced in these abstracts and papers, and we conducted a separate search for basket trials registered in ClinicalTrials.gov.

## 1.3 Data extraction

Abstracts were reviewed to assess whether the studies meet the eligibility criteria for basket trial practice. Basket trial practice includes any publication that reports on the design and findings from a basket trial. The full texts of the papers that qualified as basket trial practice were reviewed to identify unique clinical trial registry numbers (e.g., ClinicalTrials.gov NCT identifiers), and further decisions were made whether to extract data for each trial. Data relating to key characteristics of basket trials—including trial recruitment status, phase, randomization, sample size, trial duration, and other design features—were extracted independently by JH and NB using a bespoke data extraction template developed by the team prior to the beginning of literature search. Additionally, outcome data from the completed trials were independently extracted by JH and NB, and discrepancies were resolved by jointly reviewing the data. Duplicated records were identified through the NCT identifier and were removed prior to data extraction.

## 1.4 Data synthesis

We describe the basket trial practices in oncology using a narrative and descriptive statistics.

# 2. Review of Basket Trial Methods

## 2.1 Data sources and searches

The systematic searches in MEDLINE, Embase, and the Cochrane Central Register of Controlled Trials that were used for the review of basket trial practices (Table S1 – S3) were also used for the review of basket trial methods. The screening strategy focused on identifying publications of statistical methods for the design and analysis of basket trials.

## 2.2 Study inclusion and exclusion criteria

All basket trial statistical methodology papers published between January 2001 and February 2023 were considered eligible for the statistical methodology review. Three reviewers (JH, NB, and AK) independently reviewed all abstracts identified in the literature searches and assessed their eligibility for methods reviews.

## 2.3 Data extraction

Abstracts were reviewed to assess whether papers met the eligibility criteria for basket trial statistical methodology. The abstracts were screened by JH, NB, and AK to identify papers that proposed innovative statistical methodology for basket trial design and/or analysis. Papers providing systematic reviews of basket trial methods were also included. Where multiple papers referred to the same method, the first original paper that proposed the method was included as the main paper. A separate bespoke data extraction template was developed for the methods paper review, and NB and AK extracted data for each, including the type of information borrowing method used, whether the proposed method related to the design and/or analysis of basket trials, features of the method, limitations, and availability of software in the public domain.

## 2.4 Data synthesis

A narrative synthesis was used to describe the key concepts for each method, and a comparative analysis of selected basket trial methods was conducted by applying them to the actual data from selected completed basket trials. A simulation study was also conducted to assess the impact of the basket trial design on a future confirmatory trial using a hypothetical clinical development program consisting of phase Ib and phase II oncology trials.

# 3. EQUATOR Checklist

The EQUATOR checklist for the systematic review of both the basket trial practice and basket trial methods are provided in Table S4.

**Table S4: EQUATOR checklist**

| **Section and Topic** | **Item #** | **Checklist item** | **Location where item is reported** |
| --- | --- | --- | --- |
| **TITLE** | | |  |
| Title | 1 | Identify the report as a systematic review. | 1 |
| **ABSTRACT** | | |  |
| Abstract | 2 | See the PRISMA 2020 for Abstracts checklist. | 2 |
| **INTRODUCTION** | | |  |
| Rationale | 3 | Describe the rationale for the review in the context of existing knowledge. | 3-5 |
| Objectives | 4 | Provide an explicit statement of the objective(s) or question(s) the review addresses. | 4 |
| **METHODS** | | |  |
| Eligibility criteria | 5 | Specify the inclusion and exclusion criteria for the review and how studies were grouped for the syntheses. | 5 |
| Information sources | 6 | Specify all databases, registers, websites, organisations, reference lists and other sources searched or consulted to identify studies. Specify the date when each source was last searched or consulted. | 5 |
| Search strategy | 7 | Present the full search strategies for all databases, registers and websites, including any filters and limits used. | 5 |
| Selection process | 8 | Specify the methods used to decide whether a study met the inclusion criteria of the review, including how many reviewers screened each record and each report retrieved, whether they worked independently, and if applicable, details of automation tools used in the process. | 6 |
| Data collection process | 9 | Specify the methods used to collect data from reports, including how many reviewers collected data from each report, whether they worked independently, any processes for obtaining or confirming data from study investigators, and if applicable, details of automation tools used in the process. | 6 |
| Data items | 10a | List and define all outcomes for which data were sought. Specify whether all results that were compatible with each outcome domain in each study were sought (e.g. for all measures, time points, analyses), and if not, the methods used to decide which results to collect. | NA |
|  | 10b | List and define all other variables for which data were sought (e.g. participant and intervention characteristics, funding sources). Describe any assumptions made about any missing or unclear information. | Supplementary Files 2 & 3 |
| Study risk of bias assessment | 11 | Specify the methods used to assess risk of bias in the included studies, including details of the tool(s) used, how many reviewers assessed each study and whether they worked independently, and if applicable, details of automation tools used in the process. | 8 |
| Effect measures | 12 | Specify for each outcome the effect measure(s) (e.g. risk ratio, mean difference) used in the synthesis or presentation of results. | NA |
| Synthesis methods | 13a | Describe the processes used to decide which studies were eligible for each synthesis (e.g. tabulating the study intervention characteristics and comparing against the planned groups for each synthesis (item #5)). | NA |
|  | 13b | Describe any methods required to prepare the data for presentation or synthesis, such as handling of missing summary statistics, or data conversions. | NA |
|  | 13c | Describe any methods used to tabulate or visually display results of individual studies and syntheses. | NA |
|  | 13d | Describe any methods used to synthesize results and provide a rationale for the choice(s). If meta-analysis was performed, describe the model(s), method(s) to identify the presence and extent of statistical heterogeneity, and software package(s) used. | NA |
|  | 13e | Describe any methods used to explore possible causes of heterogeneity among study results (e.g. subgroup analysis, meta-regression). | NA |
|  | 13f | Describe any sensitivity analyses conducted to assess robustness of the synthesized results. | NA |
| Reporting bias assessment | 14 | Describe any methods used to assess risk of bias due to missing results in a synthesis (arising from reporting biases). | NA |
| Certainty assessment | 15 | Describe any methods used to assess certainty (or confidence) in the body of evidence for an outcome. | NA |
| **RESULTS** | | |  |
| Study selection | 16a | Describe the results of the search and selection process, from the number of records identified in the search to the number of studies included in the review, ideally using a flow diagram. | 7, Supplementary File 1 |
|  | 16b | Cite studies that might appear to meet the inclusion criteria, but which were excluded, and explain why they were excluded. | Supplementary File 1 |
| Study characteristics | 17 | Cite each included study and present its characteristics. | 9-13 , Supplementary File 2, Supplementary File 3 |
| Risk of bias in studies | 18 | Present assessments of risk of bias for each included study. | 8 |
| Results of individual studies | 19 | For all outcomes, present, for each study: (a) summary statistics for each group (where appropriate) and (b) an effect estimate and its precision (e.g. confidence/credible interval), ideally using structured tables or plots. | NA |
| Results of syntheses | 20a | For each synthesis, briefly summarise the characteristics and risk of bias among contributing studies. | NA |
|  | 20b | Present results of all statistical syntheses conducted. If meta-analysis was done, present for each the summary estimate and its precision (e.g. confidence/credible interval) and measures of statistical heterogeneity. If comparing groups, describe the direction of the effect. | NA |
|  | 20c | Present results of all investigations of possible causes of heterogeneity among study results. | NA |
|  | 20d | Present results of all sensitivity analyses conducted to assess the robustness of the synthesized results. | NA |
| Reporting biases | 21 | Present assessments of risk of bias due to missing results (arising from reporting biases) for each synthesis assessed. | NA |
| Certainty of evidence | 22 | Present assessments of certainty (or confidence) in the body of evidence for each outcome assessed. | NA |
| **DISCUSSION** | | |  |
| Discussion | 23a | Provide a general interpretation of the results in the context of other evidence. | 23-24 |
|  | 23b | Discuss any limitations of the evidence included in the review. | 23 |
|  | 23c | Discuss any limitations of the review processes used. | 23 |
|  | 23d | Discuss implications of the results for practice, policy, and future research. | 23-24 |
| **OTHER INFORMATION** | | |  |
| Registration and protocol | 24a | Provide registration information for the review, including register name and registration number, or state that the review was not registered. | NA |
|  | 24b | Indicate where the review protocol can be accessed, or state that a protocol was not prepared. | 25 |
|  | 24c | Describe and explain any amendments to information provided at registration or in the protocol. | NA |
| Support | 25 | Describe sources of financial or non-financial support for the review, and the role of the funders or sponsors in the review. | 25 |
| Competing interests | 26 | Declare any competing interests of review authors. | 25 |
| Availability of data, code and other materials | 27 | Report which of the following are publicly available and where they can be found: template data collection forms; data extracted from included studies; data used for all analyses; analytic code; any other materials used in the review. | 25 |

*From:*  Page MJ, McKenzie JE, Bossuyt PM, Boutron I, Hoffmann TC, Mulrow CD, et al. The PRISMA 2020 statement: an updated guideline for reporting systematic reviews. BMJ 2021;372:n71. doi: 10.1136/bmj.n71 For more information, visit: <http://www.prisma-statement.org/>

# 4. PRISMA Diagrams

## 4.1 PRISMA diagram for the systematic review of basket trial practices

From the systematic searches of the databases, 272 abstracts relating to basket trials practices were identified from Embase, 149 from MEDLINE, 29 from Cochrane, and 65 from ClinicalTrials.gov. After removing duplicated recodes, 365 abstracts were screened, 226 full texts were reviewed, and 146 trials were included in the review. The PRISMA diagram is presented in Figure S1.

Identification of basket trials via database search from January 2019 – February 2023

Identification

Records identified from:

Embase (n = 272)

MEDLINE (n = 149)

Cochrane (n = 29)

ClinicalTrials.gov (n = 65)

Records removed *before screening*:

Duplicate records removed

(n = 150)

Records removed due to other reasons (n = 0)

Records screened

(n = 365)

Records excluded

(n = 139)

Reports sought for retrieval

(n = 226)

Reports not retrieved

(n = 0)

Studies identified from reports and assessed for eligibility

(n = 234)

Studies excluded:

Multiple treatments (n = 38)

No binary endpoint (n = 9)

Not a basket trial / not oncology-related (n = 41)

Studies included in review

(n = 146)

Screening

Included

**Figure S1: The PRISMA diagram for systematic review of basket trial practice**

## 4.2 PRISMA diagram for the systematic review of basket trial methods

From the systematic searches of the databases, 372 abstracts relating to basket trials methods were identified from Embase and 213 from MEDLINE. After removing duplicated recodes, 468 abstracts were screened, 67 full texts were reviewed, and 41 publications were included in the review. The PRISMA diagram is presented in Figure S2.

Identification of methods papers via database search from January 2001 – February 2023

Identification

Records identified from:

Embase (n = 372)

MEDLINE (n = 213)

Cochrane (n = 36)

Records removed *before screening*:

Duplicate records removed

(n = 163)

Records removed due to other reasons (n = 0)

Records screened

(n = 468)

Records excluded

(n = 412)

Reports sought for retrieval

(n = 68)

Reports not retrieved

(n = 1)

Reports assessed for eligibility

(n = 67)

Reports excluded:

No new method proposed (n = 18)

Conference abstract, no paper with details on proposed method (n = 3)

Not a basket trial / not oncology-related (n = 5)

Reports of included methods

(n=41)

Screening

Included

**Figure S2: The PRISMA diagram for the systematic review of basket trial methods.**

# 5. Additional Results from the Systematic Review of Basket Trial Practices

## 5.1 Number of patients

Among the ongoing phase II trials that are recruiting or not yet recruiting, the average estimated number of participants was 182.1 with the smallest trial having a planned enrollment of 24 participants and the largest trial having a planned enrollment of 1609 participants. Among the completed phase II trials, the average number of participants was 138.4 with the smallest trial having 12 participants and the largest trial having 582 participants. On average, the estimated number of participants for ongoing trials is greater than the average number of participants enrolled in completed trials. One possible reason for this may be that some of the completed trials did not recruit to target, or alternatively, there could be an upward trend in the planned sample size of basket trial designs. It was not straight forward to determine the sample size for phase Ib basket trials because the total sample size, including the dose-escalation phase, was reported in most cases. The anticipated study durations for the phase II trials not yet completed ranged from 22 months to 131 months, while the actual study duration among the completed phase II trials was 23 to 122 months with an average of 60.6 months.

## 5.2 Number of tumor types, biomarkers, and prior lines of therapy

Most of the phase II basket trials investigated 2 to 5 tumor types, excluding tumor types grouped as “others”. More than 90% of the trials were in pre-treated population with at least one prior line of therapy. The number of molecular markers used to define eligibility in most basket trials was 1, with 48% (70/146) of the trials having defined eligibility criteria based on a single molecular marker. As an example of the contrary, the D-BOB basket trial (NCT04266912) investigates the best dose of avelumab in combination with M6620 in treating patients with deoxyribonucleic acid (DNA) damage repair (DDR) deficient solid tumors by enrolling participants based on the presence of actionable aberrations in one or more of the following DNA damage response (DDR) genes: ARID1A, ATM, ATR, ATRX, BAP1, BARD1, BRCA1/2, BRIP1, CDK12, CHEK2, FANCA, FANCC, FANCD2, FANCE, FANCF, FANCM, MRE11A, MSH2, NBN (NBS1), PALB2, RAD51, RAD51C, RAD51D, SMARCB1, and VHL.

## 5.3 Design and analytical methods

Two broad design approaches were mostly adopted in the phase II trials. The first design considers an adaptive or sequential approach with at least two sequential analyses. The first analysis is typically performed when a fraction of the participants has been recruited with the purpose of evaluating either the trial as a whole or individual tumor types separately for futility, and the second analysis is done at the “end-of-study” to assess efficacy of the treatment. The second type of design is a single group assignment where all participants are assigned to a cohort from the beginning of the study and assessment of efficacy is done at the end-of-study. Among the phase II trials, 38% (41/107) of the trials adopted an adaptive design, with Simon’s two stage being the most common approach. Among the 23 completed phase II trials, 11 trials (48%) calculated response rate using only the participants in the individual tumor type, while 4 trials (17%) calculated the overall response rate averaging across all patients independent of their respective tumor types. However, 6 trials (26%) calculated the response rate for each tumor type while borrowing information across tumor types using a variation of a Bayesian hierarchical model (BHM).

# 6. Comparative Analysis of Completed Trials

## 6.1 Analysis methods

### 6.1.1 Notation

Consider a trial with $K$ tumor types and let $n_{k}$ and $y_{k}$ denote the number of participants and the number of responders for the $k$th tumor type, respectively, $k=1,\ldots,K$. Further, let $\pi_{k}$ denote the $k$th tumor-specific response probability, and define

$$\theta_{k}= \log\left( \frac{\pi_{k}}{1- \pi_{k}} \right).$$

We consider the hypotheses $H_{0}: \pi_{k}\leq\pi_{0k}$ versus $H_{1}: \pi_{k}>\pi_{1k}$, where $\pi_{0k}$ and $\pi_{1k}$ denote the prespecified response rates under the null and alternative hypotheses.

### 6.1.2 Independent analyses within each tumor (i.e., no information borrowing)

We conduct independent Bayesian analyses (IND) within each tumor type where we assume the prior distributions for each tumor-specific response rate to be $\pi_{k}\sim\text{Beta}(a_{0},b_{0})$, $k=1,\ldots,K$. We choose the values of $a_{0}$ and $b_{0}$ such that

$$\frac{a_{0}}{a_{0}+b_{0}}=\frac{\pi_{0}+ \pi_{A}}{2}$$

and $a_{0}+b_{0}=1$, where $\pi_{0}= \frac{1}{K}\sum_{k=1}^{K} \pi_{0k}$ and $\pi_{A}= \frac{1}{K}\sum_{k=1}^{K} \pi_{1k}$.

### 6.1.3 Bayesian model averaging

We implement the Bayesian model averaging (BMA) approach of Psioda *et al.* [2] in which we consider all $J$ possible classifications of tumor types into subsets. A unique model is fit corresponding to each classification where tumor types within a subset are constrained to share the same response rate while differing from tumor types in other subsets.

Let $\pi_{(j,p)}|M_{j}$ denote the distinct tumor-specific response rate for the $p$th subset under model $M_{j}$, $j=1,\ldots,J$, and assume $\pi_{(j,p)}|M_{j}\sim\mathrm{Beta}(a_{0},b_{0})$ where the values of $a_{0}$ and $b_{0}$ are set equal to the hyperparameters for the IND approach. When calculating the posterior model probabilities, we set the prior probabilities for each model as

$$p\left( M_{j} \right)\propto e^{\alpha}$$

with $\alpha=2$ as recommended by Psioda *et al.* [2].

The posterior results for a given tumor type are then averaged together using BMA with the posterior model probabilities as weights.

### 6.1.4 Calibrated Bayesian hierarchical model

We fit the calibrated Bayesian hierarchical model (CBHM) proposed by Chu and Yuan [3] as

$$\theta_{1},\ldots,\theta_{K}\sim N\left( \mu, \sigma^{2} \right),$$

$$\mu\sim N\left( \mu_{0},\sigma_{0}^{2} \right).$$

For the hyperparameters, we choose $\sigma_{0}^{2}=100$ and $\mu_{0}=\sum_{k=1}^{K} \theta_{0k}$ where $\theta_{0k}$ is the logit of $\pi_{0k}$. Rather than place a prior on $\sigma^{2}$, as is traditionally done with Bayesian hierarchical models, we set

$$\sigma^{2}=\exp\left\{ a+b\times log(T) \right\},$$

where $T$ is the chi-squared test statistic for homogeneity and $a$ and $b$ are tuning parameters. Chu and Yuan [3] discuss the calibration of $a$ and $b$, which involves choosing prespecified values of the shrinkage parameter corresponding to strong information borrowing ($\sigma_{B}^{2}$) and little information borrowing ($\sigma_{\bar{B}}^{2}$). Following their example, we set $\sigma_{B}^{2}=1$ and $\sigma_{\bar{B}}^{2}=80$.

## 6.2 Results for trials with heterogeneous response rates

Table S5 shows an example of a completed basket trial with heterogeneous response rates, a cross-tumoral phase II clinical Trial exploring crizotinib in patients with advanced tumors induced by causal alterations of ALK and/or MET (NCT01524926). The trial had twelve baskets characterized by tumor types and ALK/MET alterations, each sharing a common threshold for the standard of care and a common clinical meaningful threshold. Further, this trial serves as an example of a basket trial in which the number of participants differs substantially between tumor types. The sample sizes ranged from 1 participant for ARMS (MET-/ALK-) to 40 participants for ASPS (MET+). The CBHM method resulted in similar estimated response rates compared to the independent method, an indication of a limited borrowing across baskets, whereas the BMA approach borrowed information from other baskets to a greater degree. Unlike NCT01524926, a phase Ib study of pembrolizumab in participants with select advanced solid tumors (NCT02054806) had heterogeneous response rates and a comparable sample size of around 20 participants per tumor type. Both the CBHM and the BMA approach resulted in a similar estimated response rate for anal canal SCC, biliary tract ACA, breast cancer ER+/HER2-, carcinoid tumors, and cervical SCC. Results from the analysis of completed trials with heterogeneous response rates are presented in Table S5.

**Table S5: Results of basket trial design with heterogeneous response rates**

|  | Observed Data | | |  | Trial Criteria | |  | Estimated ORR (Posterior Probability) | | |
| --- | --- | --- | --- | --- | --- | --- | --- | --- | --- | --- |
| Tumor Type/Basket | $\#$  Participants | $\#$  Responders | Response Rate |  | SoC  ORR | Targeted ORR |  | IND | BMA | CBHM |
| ClinicalTrials.gov ID: NCT01848834 |  |  |  |  |  |  |  |  |  |  |
| Triple negative breast cancer | 32 | 5 | 0.156 |  | 0.20 | 0.45 |  | 0.159 (0.242) | 0.171 (0.266) | 0.182 (0.283) |
| Head & neck cancer | 60 | 10 | 0.167 |  | 0.10 | 0.35 |  | 0.168 (0.940) | 0.174 (0.970) | 0.182 (0.998) |
| Urothelial cancer | 33 | 7 | 0.212 |  | 0.10 | 0.35 |  | 0.214 (0.968) | 0.200 (0.983) | 0.186 (0.998) |
| Gastric cancer | 39 | 8 | 0.205 |  | 0.15 | 0.40 |  | 0.207 (0.809) | 0.197 (0.825) | 0.185 (0.858) |
| ClinicalTrials.gov ID: NCT01970540 |  |  |  |  |  |  |  |  |  |  |
| SCLC 2nd line | 28 | 10 | 0.357 |  | 0.10* | 0.30* |  | 0.352 (1.000) | 0.364 (1.000) | 0.371 (1.000) |
| Endometrial | 19 | 8 | 0.421 |  | 0.10* | 0.30* |  | 0.410 (1.000) | 0.397 (1.000) | 0.401 (1.000) |
| ClinicalTrials.gov ID: NCT02454972 |  |  |  |  |  |  |  |  |  |  |
| Biliary tract carcinoma | 18 | 1 | 0.056 |  | 0.01 | 0.10 |  | 0.057 (0.864) | 0.072 (0.928) | 0.056 (0.840) |
| Unknown primary site | 19 | 0 | 0.000 |  | 0.01 | 0.10 |  | 0.004 (0.107) | 0.010 (0.201) | 0.000 (0.000) |
| Endometrial | 71 | 8 | 0.113 |  | 0.10 | 0.25 |  | 0.112 (0.596) | 0.109 (0.565) | 0.113 (0.597) |
| Ewings | 28 | 4 | 0.143 |  | 0.01 | 0.10 |  | 0.141 (1.000) | 0.132 (1.000) | 0.143 (1.000) |
| Germ cell | 23 | 1 | 0.043 |  | 0.01 | 0.10 |  | 0.045 (0.827) | 0.060 (0.902) | 0.043 (0.802) |
| Head & neck | 13 | 0 | 0.000 |  | 0.01 | 0.10 |  | 0.006 (0.131) | 0.014 (0.251) | 0.000 (0.000) |
| Metastatic breast carcinoma | 21 | 6 | 0.286 |  | 0.01 | 0.10 |  | 0.277 (1.000) | 0.288 (1.000) | 0.285 (1.000) |
| Neuroendocrine | 31 | 2 | 0.065 |  | 0.01 | 0.10 |  | 0.065 (0.968) | 0.076 (0.983) | 0.065 (0.962) |
| Small cell lung cancer | 105 | 38 | 0.362 |  | 0.15 | 0.3 |  | 0.359 (1.000) | 0.352 (1.000) | 0.362 (1.000) |
| ClinicalTrials.gov ID: NCT02034110 |  |  |  |  |  |  |  |  |  |  |
| High-grade glioma cohort | 45 | 15 | 0.333 |  | 0.10 | 0.50 |  | 0.333 (1.000) | 0.351 (1.000) | 0.334 (1.000) |
| Low-grade glioma cohort | 13 | 7 | 0.538 |  | 0.10 | 0.50 |  | 0.523 (1.000) | 0.520 (1.000) | 0.539 (1.000) |
| Biliary tract cancer | 43 | 22 | 0.512 |  | 0.10 | 0.50 |  | 0.507 (1.000) | 0.507 (1.000) | 0.512 (1.000) |
| Anaplastic thyroid cancer | 16 | 11 | 0.688 |  | 0.15 | 0.60 |  | 0.666 (1.000) | 0.644 (1.000) | 0.687 (1.000) |
| Hairy cell leukemia | 24 | 22 | 0.917 |  | 0.10 | 0.50 |  | 0.893 (1.000) | 0.883 (1.000) | 0.916 (1.000) |
| ClinicalTrials.gov ID: NCT01524926 |  |  |  |  |  |  |  |  |  |  |
| CCSA (MET+) | 26 | 1 | 0.038 |  | 0.10 | 0.30 |  | 0.044 (0.092) | 0.054 (0.095) | 0.039 (0.072) |
| CCSA (MET-) | 2 | 0 | 0.000 |  | 0.10 | 0.30 |  | 0.067 (0.204) | 0.127 (0.359) | 0.000 (0.000) |
| IMFT (ALK+) | 12 | 6 | 0.500 |  | 0.10 | 0.30 |  | 0.477 (1.000) | 0.395 (0.999) | 0.501 (0.999) |
| IMFT (ALK-) | 7 | 1 | 0.143 |  | 0.10 | 0.30 |  | 0.150 (0.575) | 0.151 (0.483) | 0.142 (0.529) |
| ASPS (MET+) | 40 | 1 | 0.025 |  | 0.10* | 0.30* |  | 0.029 (0.023) | 0.047 (0.044) | 0.025 (0.016) |
| ASPS (MET-) | 4 | 1 | 0.250 |  | 0.10* | 0.30* |  | 0.240 (0.749) | 0.234 (0.681) | 0.251 (0.729) |
| ASPS (MET?) | 1 | 0 | 0.000 |  | 0.10* | 0.30* |  | 0.100 (0.272) | 0.155 (0.428) | 0.000 (0.000) |
| PRCC1 (MET+) | 4 | 2 | 0.500 |  | 0.10 | 0.30 |  | 0.440 (0.967) | 0.358 (0.943) | 0.498 (0.972) |
| PRCC1 (MET-) | 16 | 1 | 0.063 |  | 0.10 | 0.30 |  | 0.071 (0.246) | 0.072 (0.199) | 0.063 (0.208) |
| PRCC1 (MET?) | 3 | 1 | 0.333 |  | 0.10 | 0.30 |  | 0.300 (0.815) | 0.270 (0.754) | 0.334 (0.815) |
| ARMS (MET+/ALK-) | 7 | 1 | 0.143 |  | 0.10 | 0.30 |  | 0.150 (0.575) | 0.151 (0.483) | 0.143 (0.528) |
| ARMS (MET-/ALK-) | 1 | 0 | 0.000 |  | 0.10 | 0.30 |  | 0.100 (0.272) | 0.155 (0.428) | 0.000 (0.000) |
| ClinicalTrials.gov ID: NCT02054806 |  |  |  |  |  |  |  |  |  |  |
| Anal canal SCC | 25 | 4 | 0.160 |  | 0.10 | 0.35 |  | 0.162 (0.803) | 0.152 (0.827) | 0.160 (0.784) |
| Biliary tract ACA | 23 | 4 | 0.174 |  | 0.10 | 0.35 |  | 0.176 (0.842) | 0.157 (0.854) | 0.174 (0.831) |
| Breast cancer ER+ HER2- | 25 | 3 | 0.120 |  | 0.10 | 0.35 |  | 0.124 (0.594) | 0.134 (0.704) | 0.120 (0.566) |
| Carcinoid tumors | 25 | 3 | 0.120 |  | 0.10* | 0.35* |  | 0.124 (0.594) | 0.134 (0.704) | 0.120 (0.562) |
| Cervical SCC | 24 | 4 | 0.167 |  | 0.10 | 0.35 |  | 0.169 (0.823) | 0.155 (0.841) | 0.166 (0.810) |
| Colon/rectal ACA | 23 | 1 | 0.043 |  | 0.10 | 0.35 |  | 0.051 (0.129) | 0.109 (0.526) | 0.043 (0.094) |
| Endometrial carcinoma | 23 | 3 | 0.130 |  | 0.10 | 0.35 |  | 0.134 (0.648) | 0.139 (0.737) | 0.131 (0.621) |
| Esophageal SCC/ACA | 23 | 7 | 0.304 |  | 0.10 | 0.35 |  | 0.301 (0.996) | 0.180 (0.984) | 0.304 (0.995) |
| GBM | 25 | 2 | 0.080 |  | 0.10 | 0.35 |  | 0.086 (0.329) | 0.118 (0.587) | 0.080 (0.292) |
| Leiomyosarcoma | 24 | 1 | 0.042 |  | 0.10* | 0.35* |  | 0.049 (0.117) | 0.108 (0.519) | 0.042 (0.089) |
| Mesothelioma (MSM) | 25 | 5 | 0.200 |  | 0.10 | 0.35 |  | 0.201 (0.922) | 0.166 (0.913) | 0.199 (0.911) |
| Nasopharyngeal carcinoma | 27 | 7 | 0.259 |  | 0.10 | 0.35 |  | 0.258 (0.989) | 0.178 (0.976) | 0.258 (0.988) |
| Neuroendocrine carcinomas | 16 | 1 | 0.063 |  | 0.10* | 0.35* |  | 0.072 (0.254) | 0.120 (0.596) | 0.062 (0.204) |
| Ovarian epithelial FTC/PPC | 26 | 3 | 0.115 |  | 0.10 | 0.35 |  | 0.119 (0.568) | 0.132 (0.688) | 0.115 (0.535) |
| Pancreatic ACA | 24 | 0 | 0.000 |  | 0.10 | 0.35 |  | 0.009 (0.007) | 0.103 (0.480) | 0.002 (0.001) |
| Prostate ACA | 23 | 4 | 0.174 |  | 0.10 | 0.35 |  | 0.176 (0.842) | 0.157 (0.854) | 0.173 (0.829) |
| Salivary gland carcinoma | 26 | 3 | 0.115 |  | 0.10 | 0.35 |  | 0.119 (0.568) | 0.132 (0.688) | 0.115 (0.532) |
| SCLC | 24 | 8 | 0.333 |  | 0.10 | 0.35 |  | 0.329 (0.999) | 0.181 (0.991) | 0.333 (0.999) |
| Thyroid cancer | 22 | 2 | 0.091 |  | 0.10 | 0.35 |  | 0.097 (0.404) | 0.123 (0.626) | 0.091 (0.364) |
| Vulvar SCC | 18 | 1 | 0.056 |  | 0.10* | 0.35* |  | 0.064 (0.210) | 0.116 (0.572) | 0.056 (0.168) |

**Trial criteria values not reported in trial publications, values instead assumed by study authors for the comparative analysis.*

*Note that the posterior probability is* $P(ORR > {ORR}_{SoC}|Data)$*.*

# 7. Simulation Setting: Technical Information

We design a simulation study for a hypothetical clinical development plan with a dose expansion phase Ib basket trial followed by phase II randomized clinical trials (RCT)—a separate phase II trial for each tumor type in which signal was detected from phase Ib —that compare treatment to control. Both trials have a binary endpoint.

## 7.1 Study design characteristics

To simplify the simulation study, we consider the following design characteristics:

- $K=5$ tumor types considered in the phase Ib trial
- Fixed and equal sample size of $N_{1}=10$ for each tumor type in the phase Ib trial (simulation study repeated with $N_{1}=20$ for each tumor type, with results presented in Section 8 of Supplementary File 1)
- Phase II sample sizes per arm ($N_{2}$) ranging between 60 and 200 in increments of 5, with the additional inclusion of $N_{2}=62$ (chosen to ensure at least 80% power to detect a risk difference between 10% and 30% in a phase II trial with a one-sided significance level of 0.025)
- An interim analysis to test for futility in the phase II trial with $N_{2,I}=\text{ceiling}\left( \frac{N_{2}}{2} \right)$ participants per arm
- No differences in accrual rate and no dropouts for either trial (i.e., simulate all final analysis data together)

## 7.2 Simulation parameters

Suppose we wish to test $H_{0}: \pi_{k}\leq\pi_{0}$ vs. $H_{1}: \pi_{k}> \pi_{0}$ for each tumor type in the phase Ib trial where $\pi_{k}$ denotes the $k$th tumor-specific response rate ($k=1,\ldots, K$) and $\pi_{0}$ denotes the response rate for the standard of care (SoC) or a meaningful clinical threshold. Further, let $\pi_{A}$ denote the response rate corresponding to clinical benefit, and suppose we set $\pi_{0}=0.10$ and $\pi_{A}=0.30$.

We vary the underlying tumor-specific response rates $\pi_{k}$ by considering six scenarios in which tumor types are specified to be either active ($\pi_{k}=\pi_{A}$) or inactive ($\pi_{k}=\pi_{0}$). The six scenarios are defined in Table S6.

**Table S6: Scenarios for simulation study as defined by underlying tumor-specific response rates.**

|  | Underlying Tumor-Specific Response Rate ($\pi_{k}$) | | | | |
| --- | --- | --- | --- | --- | --- |
| Scenario | Tumor Type 1 | Tumor Type 2 | Tumor Type 3 | Tumor Type 4 | Tumor Type 5 |
| 1 | $\pi_{0}$ | $\pi_{0}$ | $\pi_{0}$ | $\pi_{0}$ | $\pi_{0}$ |
| 2 | $\pi_{A}$ | $\pi_{0}$ | $\pi_{0}$ | $\pi_{0}$ | $\pi_{0}$ |
| 3 | $\pi_{A}$ | $\pi_{A}$ | $\pi_{0}$ | $\pi_{0}$ | $\pi_{0}$ |
| 4 | $\pi_{A}$ | $\pi_{A}$ | $\pi_{A}$ | $\pi_{0}$ | $\pi_{0}$ |
| 5 | $\pi_{A}$ | $\pi_{A}$ | $\pi_{A}$ | $\pi_{A}$ | $\pi_{0}$ |
| 6 | $\pi_{A}$ | $\pi_{A}$ | $\pi_{A}$ | $\pi_{A}$ | $\pi_{A}$ |

Scenarios 2-5 (heterogeneous response rates) are motivated by the completed trial NCT01885195 (three tumor types, one with observed ORR of 0.03 and two with ORRs of 0.33), whereas scenario 6 (homogeneous active response rates) is motivated by the completed trial NCT01631552 (three tumor types, each with observed objective response rates, or ORRs, between 0.29-0.33).

## 7.3 Statistical analysis methods for phase Ib trial

For the phase Ib trial, we calculate posterior probabilities for each tumor-specific response rate $\pi_{k}$ using two methods:

- Independent Bayesian analyses (IND) within each tumor type (i.e., no information borrowing)
- Bayesian model averaging (BMA) approach (Psioda *et al.* [2])

For both methods, we use the same choices for prior distributions as described in Section 6 “Comparative Analysis of Completed Trials” of Supplementary File 1.

## 7.4 Posterior probabilities and success criteria

We consider a case in which any tumor type with detectable signal in phase Ib is further investigated with a separate phase II trial (i.e., for each simulated phase Ib dataset, 0 to 5 subsequent phase II trials will follow depending on the number of tumor types identified to have detectable signal). For a given phase II trial corresponding to the $k$th tumor type, we derive posterior distributions with respect to both the response rate in the treatment group ($\pi_{k}$) and the response rate in the control group ($\pi_{C,k}$), where we specify a $\text{Beta}(0.2, 0.8)$ prior for both parameters.

Let $\boldsymbol{D}_{\boldsymbol{1}}$ denote the observed data from the phase Ib trial. We consider the $k$th tumor type in the phase Ib trial to be successful (i.e., have detectable signal) if

$$\Pr\left( \pi_{k}> \pi_{0} \right|\boldsymbol{D}_{\boldsymbol{1}})\geq0.80.$$

Similarly, let $\boldsymbol{D}_{\boldsymbol{2,I,k}}$ and $\boldsymbol{D}_{\boldsymbol{2,F,k}}$ denote the observed phase II data at the time of the interim and final analyses, respectively, for tumor type $k$. At interim, we stop the trial for futility if

$$\Pr\left( \pi_{k}-\pi_{C,k}> 0 \right|\boldsymbol{D}_{\boldsymbol{2,I,k}})\leq0.10,$$

and we declare success at the end of the trial if

$$\Pr\left( \pi_{k}-\pi_{C,k}> 0 \right|\boldsymbol{D}_{\boldsymbol{2,F,k}})\geq0.90.$$

## 7.5 General simulation setup

For each of the six defined scenarios, we repeat the following steps $B=100,000$ times:

*Simulate data:*

1. For phase Ib data, simulate the number of responders $y_{1k}\sim\text{Binomial}(N_{1},\pi_{k})$ for tumor type $k$, $k=1,\ldots,K$, and set $\boldsymbol{D}_{\boldsymbol{1}}=\{y_{11},\ldots, y_{1K}\}$.
2. For phase II interim data corresponding to tumor type $k$, $k=1,\ldots,K$, simulate the number of responders in the treatment group and the number of responders in the control group as $y_{T,I,k}\sim\text{Binomial}(N_{2,I},\pi_{k})$ and $y_{C,I,k}\sim\text{Binomial}(N_{2,I},\pi_{C})$, respectively, where $\pi_{k}$ is the same underlying response rate for tumor type $k$ from the phase Ib trial and $\pi_{C}=0.10$, and set $\boldsymbol{D}_{\boldsymbol{2,I,k}}=\{y_{T,I,k}, y_{C,I,k}\}$. Similarly, simulate the post-interim data for both arms as $y_{T,F,k}\sim\text{Binomial}(N_{2,I},\pi_{k})$ and $y_{C,F,k}\sim\text{Binomial}(N_{2,I},\pi_{C})$, and set $\boldsymbol{D}_{\boldsymbol{2,F,k}}=\{y_{T,I,k}, y_{C,I,k}, y_{T,F,k}, y_{C,F,k}\}$.

*Phase Ib analyses:*

1. For the phase Ib trial, calculate $\Pr\left( \pi_{k}> \pi_{0} \right|\boldsymbol{D}_{\boldsymbol{1}})$ for each tumor type using the IND approach, and define an indicator of success for each tumor type; i.e., $\Phi_{IND,1,k}^{\left( b \right)}=I\left[ \Pr\left( \pi_{k}> \pi_{0} \right|\boldsymbol{D}_{\boldsymbol{1}})\geq0.80 \right]$, $k=1,\ldots,K$, where $I[\cdot]$ is the indicator function.
2. Repeat step 3 using the BMA approach to obtain posterior probabilities on each of the tumor-specific response rates, and denote the indicators of success for each tumor type by $\Phi_{BMA,1,k}^{\left( b \right)}$, $k=1,\ldots,K$.

*Phase II analyses:*

1. For the phase II interim analysis for tumor type $k$, $k=1,\ldots,K$, calculate $\Pr\left( \pi_{k}-\pi_{C,k}>0 \right|\boldsymbol{D}_{\boldsymbol{2,I,k}})$ and set $\Phi_{2,k}^{(b)}=0$ if $\Pr\left( \pi_{k}-\pi_{C,k}>0 \right|\boldsymbol{D}_{\boldsymbol{2,I,k}})\leq0.10$. Otherwise, proceed to step 6.
2. For the phase II final analysis for tumor type $k$, calculate $\Pr\left( \pi_{k}-\pi_{C,k}> 0 \right|\boldsymbol{D}_{\boldsymbol{2,F,k}})$ and define the indicator $\Phi_{2,k}^{\left( b \right)}=I\left[ \Pr\left( \pi_{k}-\pi_{C,k}> 0 \right|\boldsymbol{D}_{\boldsymbol{2,F,k}})\geq0.90 \right]$.

After repeating steps 1-6 $B$ times, we calculate the phase Ib probability of success (PoS) and the joint PoS (success in both phase Ib and phase II) for each tumor type under the two analysis approaches:

- Phase Ib PoS for the $k$th tumor type, $k=1,\ldots,K$
  - IND approach: $\text{PoS}_{IND,1,k}=\frac{1}{B}\sum_{b=1}^{B} \Phi_{IND,1,k}^{\left( b \right)}$
  - BMA approach: $\text{PoS}_{BMA,1,k}=\frac{1}{B}\sum_{b=1}^{B} \Phi_{BMA,1,k}^{\left( b \right)}$
- Joint PoS for the $k$th tumor type, $k=1,\ldots,K$, for each phase Ib analysis approach
  - IND approach: $\text{PoS}_{IND,joint,k}=\frac{1}{B}\sum_{b=1}^{B} \left( \Phi_{IND,1,k}^{\left( b \right)}\times\Phi_{2,k}^{\left( b \right)} \right)$
  - BMA approach: $\text{PoS}_{BMA,joint,k}=\frac{1}{B}\sum_{b=1}^{B} \left( \Phi_{BMA,1,k}^{\left( b \right)}\times\Phi_{2,k}^{\left( b \right)} \right)$

In addition to calculating the phase Ib PoS and joint PoS for each tumor type, we calculate the mean squared error (MSE) when estimating each tumor-specific response rate in phase Ib under both analysis approaches. Let $\hat{\pi}_{IND,k}$ and $\hat{\pi}_{BMA,k}$ denote the estimated posterior means of $\pi_{k}$ under the IND approach and the BMA approach, respectively, in phase Ib. We calculate the MSE as follows:

- IND approach: $\text{MSE}_{IND,k}= \frac{1}{B}{\sum_{b=1}^{B} \left( \hat{\pi}_{IND,k}-\pi_{k} \right)}^{2}$
- BMA approach: $\text{MSE}_{BMA,k}= \frac{1}{B}{\sum_{b=1}^{B} \left( \hat{\pi}_{BMA,k}-\pi_{k} \right)}^{2}$

# 8. Additional Simulation Results

## 8.1 Simulation results with a phase Ib sample size of 10 per tumor type

Figure S3 shows the advantages of information borrowing methods when estimating the tumor-specific ORRs with greater quality as measured via mean squared error (MSE). The BMA approach resulted in lower MSE in each scenario when estimating the ORR for active tumor types and for two of the five scenarios when estimating the ORR for inactive tumor types.

***
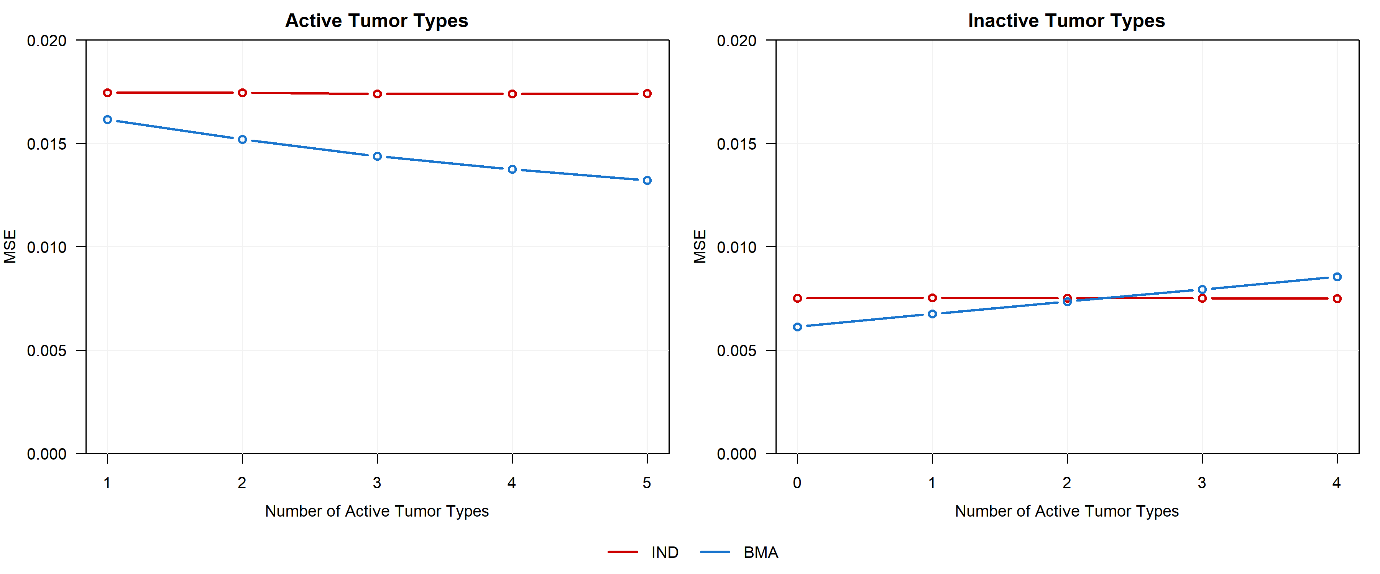
***

**Figure S3: MSE when estimating the ORR of active tumor types (left panel) and inactive tumor types (right panel) in phase Ib across scenarios defined by the number of active tumor types (ORR of 30%) and inactive tumor types (ORR of 10%). Each tumor type has a sample size of 10 participants.**

## 8.2 Simulation results with a phase Ib sample size of 20 per tumor type

We repeated the simulation study described in Section 3.4 of the manuscript with the only change being the increase of the phase Ib sample size from 10 to 20 per tumor type. The phase Ib and joint probability of success (PoS), the relationship between cost (i.e., planned phase II sample size) and PoS, and the MSE when estimating tumor-specific ORRs in phase Ib are shown in Figures S4, S5, and S6, respectively.


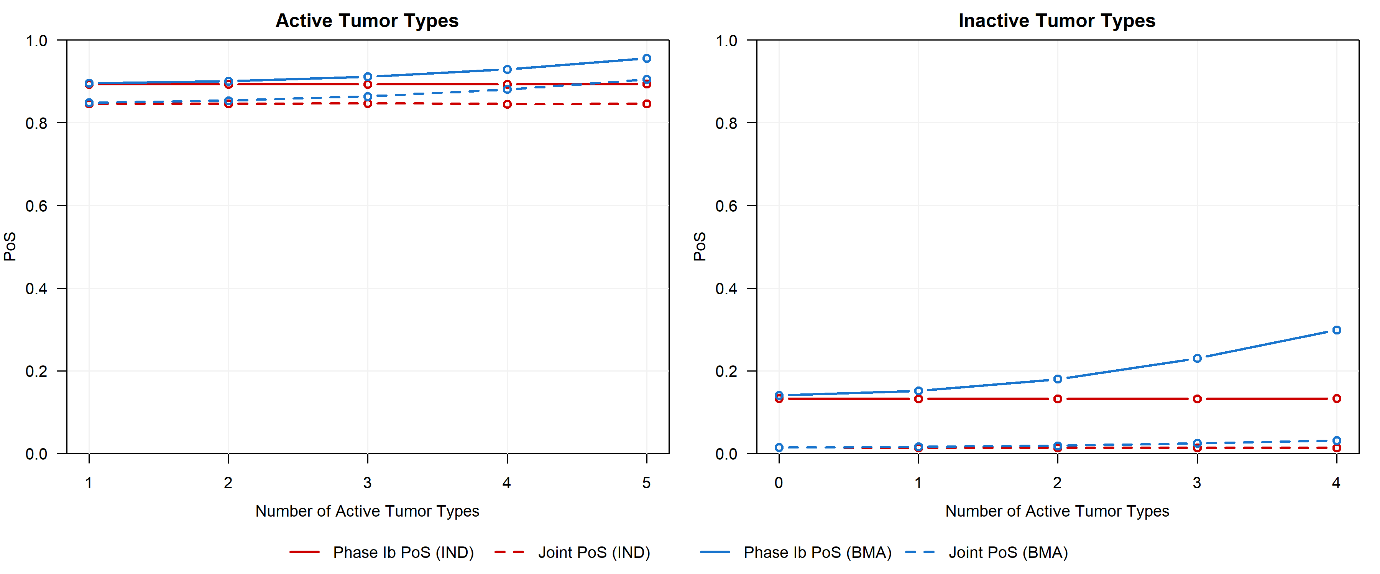


**Figure S4: *Phase Ib PoS and joint PoS for active tumor types (left panel, ORR of 30%) and inactive tumor types (right panel, ORR of 10%) across various scenarios that differ in the number of active tumor types. Each tumor type has a sample size of 20 participants.***


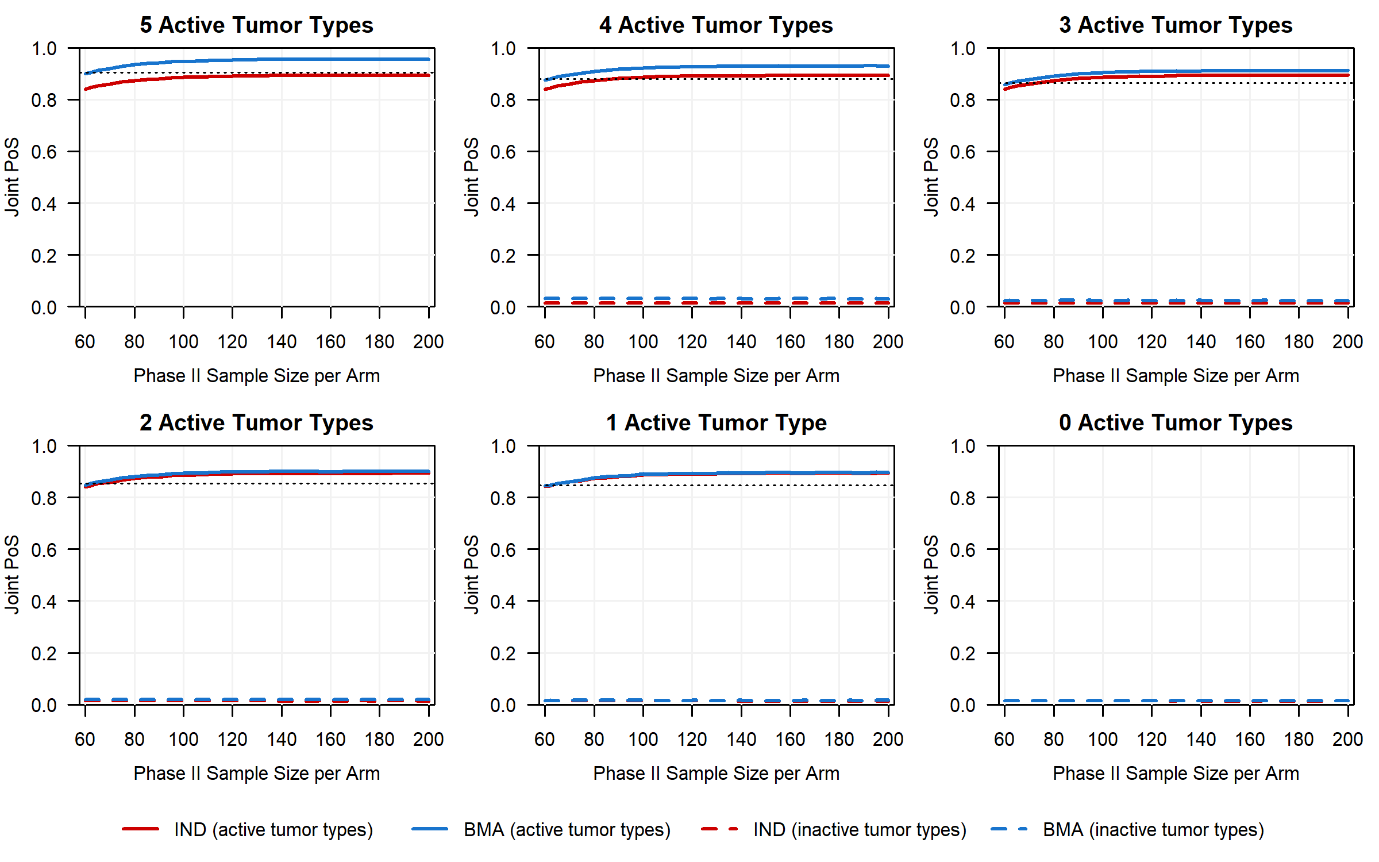


**Figure S5: *Planned phase II sample size per arm versus the joint PoS for all six scenarios in which we range the number of active tumor types (ORR of 30%) between 0 and 5. The black horizontal dashed line for the first five scenarios corresponds to the joint PoS obtained for active baskets if using the BMA approach in phase Ib and a planned phase II sample size of 62 per arm (i.e., sample size chosen to ensure at least 80% power to detect a risk difference of 20% in a phase II trial). Each tumor type has a sample size of 20 participants.***


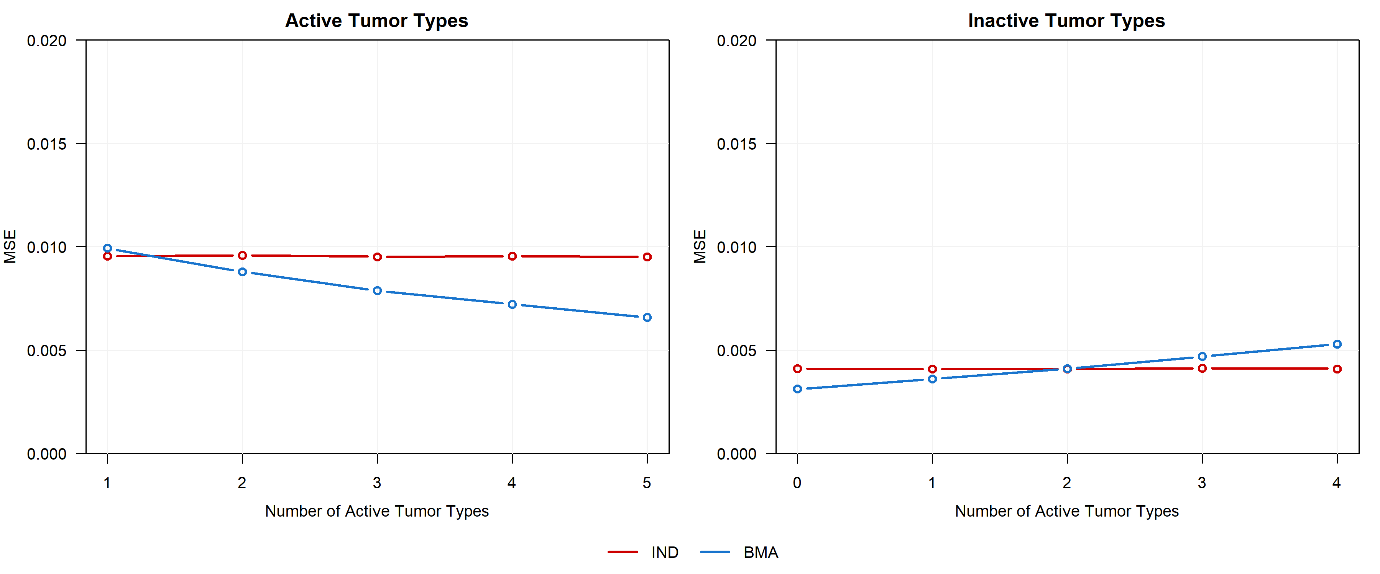


**Figure S6: *MSE when estimating the ORR of active tumor types (left panel) and inactive tumor types (right panel) in phase Ib across scenarios defined by the number of active tumor types (ORR of 30%) and inactive tumor types (ORR of 10%). Each tumor type has a sample size of 20 participants.***

# 9. Systematic Review Protocol

## 9.1 Introduction

Advancement in genomics technology has enabled innovation in oncology drug development over the last decade. There has been growing interest in precision-based medicine, which aims to improve the treatment of disease by identifying therapies that can specifically affect disease targets based on their genetic make-up. Innovation in biotechnology, matched with advanced computational tools, provide deeper insights about molecular drug-able genomic targets to cure or slow down cancer progression. A paradigm shift from systemic chemotherapy to targeted therapies has generated new hypothesis as to whether a drug is specific to a cancer site or has tissue-agonistic effect. A targeted therapy such as anti-PD-L1 should in principle work across cancer indications if the genomic target or predictive biomarker is present. In contrary to the expected biological mechanism of tissue agonistic agents, substantial variations are observed between cancer indications, and the hypothesis of whether an agent is tissue-agonistic or specific to a cancer site/indication remains an ongoing challenge in oncology drug development. To secure tissue-agnostic approvals, drug developers need to be able to generalize treatment effects observed in some cancers to other cancers with the same molecular alterations. Examples of approved tissue-agonistic drugs are dostarlimab (GSK), pembrolizumab (Merck), and larotrectinib (Eli Lilly).

Master protocols are being adopted by many pharmaceutical companies and cancer research institutes to increase both the scale and pace of oncology drug development. They have the potential to support smart risk taking as to whether a new drug holds promise to progress to expensive late stages of clinical development. The basket trial design is more amenable for designing and evaluating proof of concept studies at pace and scale and for evaluating whether a molecular therapy is tissue-agonistic or specific to cancer site or indication. Unlike other master protocols, the basket trial design aims to evaluate efficacy and safety of a single molecular therapy in multiple cancer indications. It is defined as any prospective clinical trial that tests the utility (e.g., effectiveness, dosage, and safety) of intervention(s) in a study population of multiple diseases with common predictive biomarkers and/or other common predictive patient characteristics that can be used to predict whether a patient will respond to a specific intervention as the unifying eligibility criteria. Although the number of basket trials in oncology drug development has increased drastically in recent years, few drugs have been approved for tissue-agonistic effect. The basket trial design can be with or without a control group, as shown in Figure S7.

Basket trial design presents statistical challenges on how to evaluate the hypothesis of tissue-agonistic effect for a new targeted therapy. Statistical challenges are exacerbated by unequal sample size and representations of the different cancer indications. As a result, several statistical methods have been developed to efficiently estimate treatment effect in a basket trial design. These methods can be broadly classified into indication-specific analysis, pooled effect, and analyses with information borrowing. However, there is lack of comparative analysis of the methods on real-life data to catalogue their strengths and limitations.

This project aims to guide design and analysis of basket trial in clinical development of oncology drugs. It consists of two parts: the first part will focus on basket trial practice to complement existing literature syntheses on basket trials by reviewing only the newest information on key design features, disease area features, trial operational challenges, and data from basket trials in oncology drug development. This part of the review will build on the previous review by Park *et al.* [1], which was discussed by Hobbs *et al.* [5]. The second part aims to review statistical analysis methods of basket trial design, focusing on the key features of the different methods and whether each method is readily accessible based on availability of software for design and analysis.

**Figure S7: Illustration of basket trial design with and without a control group (Source: Park et al. [4])**

## 9.2 Methods and analysis

### 9.2.1 External search strategy: basket trial practice

To maximize efficiency and avoid duplication, this systematic review of basket trial practice was designed as an update of a prior review for which searches were carried out in July 2019 (Park *et al.* [1]). Our search strategy thus mirrored the search strategy of Park *et al.* [1] with minor modifications. Search terms were modified to focus only on basket trials. We will search MEDLINE, Embase, and the Cochrane Central Register of Controlled Trials to identify eligible studies and trials. The search will be supplemented with a review of bibliographies from included publications and trial registries (ClinicalTrials.gov and ISRCTN registry) for registered basket trial protocols.

**Table S7: Search terms for basket trial design and practice**

| **No.** | **Terms** | **Hits** | **Comments** |
| --- | --- | --- | --- |
| 1 | Basket trial or basket clinical trial or adaptive basket trial or basket adaptive trial |  |  |
| 2 | Adaptive clinical trials |  |  |
| 3 | Adaptive clinical trial |  |  |
| 4 | Adaptive design |  |  |
| 5 | Flexible trial design |  |  |
| 6 | Basket trial analysis |  |  |
| 7 | Enrichment design or population enrichment or population enrichment design or Bayesian adaptive enrichment) |  |  |
| 8 | Biomarker design or biomarker adaptive or biomarker adaptive design or biomarker adjusted) |  |  |
| 9 | Seamless design or adaptive seamless or operationally seamless or operational seamless or inferentially seamless or inferential seamless |  |  |
| 10 | "Seamless 2-3" or "seamless 2 to 3" or "seamless 2/3" or "seamless phase ii/iii" or "seamless phase 2/3" |  |  |
| 11 | "Seamless 1-2" or "seamless 1 to 2" or "seamless 1/2" or "seamless phase i/ii" or "seamless phase 1/2" |  |  |
| 12 | or/1-11 |  |  |
| 13 | Humans/ |  |  |
| 14 | Animals/ |  |  |
| 15 | (12 and 13) not 14 |  |  |
| 16 | limit 15 to English language |  |  |

### 9.2.2 External search strategy: statistical methods

A search of MEDLINE, Embase, and the Cochrane Central Register of Controlled Trials will be done to identify statistical methods for analyzing data from basket trials. The search will focus on new methods for basket trial analysis as well as application of existing methods from January 2000 to date.

**Table S8: Search terms for statistical methods for analyzing basket trials**

| **No.** | **Terms** | **Hits** | **Comments** |
| --- | --- | --- | --- |
| 1 | (Basket trial or basket clinical trial or adaptive basket trial or basket adaptive trial).mp. |  |  |
| 2 | Statistical method |  |  |
| 3 | Data science |  |  |
| 4 | Machine learning |  |  |
| 5 | AI |  |  |
| 6 | Artificial intelligence |  |  |
| 7 | or/2-6 |  |  |
| 8 | software |  |  |
| 9 | R package |  |  |
| 10 | Code |  |  |
| 11 | Shiny App |  |  |
| 12 | or/8-11 |  |  |
| 13 | (1 and 7 and 12 ) |  |  |
| 16 | limit 13 to English language |  |  |

### 9.2.3 Internal GSK search strategy

In additional to external database search, we will search an internal GSK database to identify past trials that adopted basket trials as a master protocol or individual application of the same targeted therapy on different cancer indications focusing on the same genomic target.

**Table S9: Search terms for internal GSK database**

| **No.** | **Terms** | **Hits** | **Comments** |
| --- | --- | --- | --- |
| 1 | Oncology |  |  |
| 2 | Phase 2 or PH2 or Ph II |  |  |
| 3 | Targeted therapy |  |  |
| 4 | (1 and 2 and 3) |  |  |

### 9.2.4 Study inclusion and exclusion criteria

Peer-reviewed publications, conference abstracts, and clinical registry records reporting on basket trials that have been proposed, are ongoing, or have already been conducted will be included in the review. We define “basket trials” as per Park *et al*. [1] as any prospective clinical trials that investigated the utility (e.g., effectiveness, dosage, and safety) of intervention(s) in a study population of multiple diseases with common predictive biomarkers or other common predictive patient characteristics that can be used to predict whether a patient will respond to a specific intervention (or both) as the unifying eligibility criteria. For the systematic review of methods for analysing basket trials, all peer-reviewed published papers or in press will be considered eligible for this review. The eligible papers, protocols, and abstract are restricted to English language only.

Two reviewers (NB and JH) will independently review all abstracts and proceedings identified in the literature searches. The full-text publications of potentially relevant abstracts will then be retrieved and assessed for eligibility. The same two reviewers will also screen the bibliographies of published literature reviews on basket trial protocols and trial registries. Discrepancies in study selection will be resolved by discussion or, when necessary, by a third investigator (AK) or the wider team including MP and HZ. Eligibility criteria are presented in Table 4.

**Table S10: PICOS (population, intervention, comparator, outcomes, study design) criteria**

| Category | Inclusion criteria |
| --- | --- |
| Population | Humans |
| Interventions | No restrictions |
| Comparator | No restrictions |
| Outcomes | No restrictions |
| Study design | Basket trial design |
| Other | Peer-reviewed publications and conference abstracts with results or published protocols in the English language |

### 9.2.5 Data extraction

The studies retrieved during the search will be screened for eligibility, and those identified as being potentially eligible will be fully assessed against the inclusion/exclusion criteria. Data from the eligible studies will be extracted using a bespoke data extraction template to be developed by the team prior to the beginning of the literature search. Study design elements, patient characteristics, and outcomes will be extracted independently by two investigators (NB and JH) using the bespoke data extraction template. Information on trial registry, trial recruitment status, phase, randomization, masking, number of clinical centres, sample size, trial duration, interventions and control, disease area, age of population, number of conventional diseases recruited, key eligibility for stratification, number of subgroups defined, and geographic location of the basket trials will be recoded. In addition, data from completed studies will also be extracted for comparative analysis of statistical methods. Data extraction will include both new trials identified in our search from 2019 to date and trials that were identified by Park *et al*. [1] but were not completed prior to 2019. Basket trials that were not completed during the review by Park *et al*. [1] will be reviewed to obtain updated data or results.

A separate bespoke data extraction template will be developed for the systematic review of statistical methods for basket trial design and/or analysis. The template will capture key features of the method, whether it is compared with another method or not, and limitations as per the respective authors. Discrepancies will be resolved by discussion or adjudication by AK or the wider team including MP and HZ.

### 9.2.6 Risk of bias

The systematic literature review will be designed in accordance with the Preferred Reporting Items for Systematic Reviews and Meta-Analysis (PRISMA) guidelines. In addition, a quality assessment will be carried out using the Cochrane Collaboration's risk of bias tool (Higgins JPT, Green S. The Cochrane Handbook for Systematic Reviews of Interventions version 5.1.0. The Cochrane Collaboration, 2011. http://handbook.cochrane.org) tool. Two reviewers (NB and JH) will conduct the assessment, to ensure consistency and to minimize individual bias.

### 9.2.7 Data synthesis and analysis

A narrative synthesis will be used to present the current landscape of basket trial in oncology based on key features including cancer indications, type of treatment, number of baskets, phase of trial, type of study (randomized vs single arm), and type of endpoints (objective response rate or others), and stage of study (with interim analysis or not). The narrative synthesis will be done under four broad categories; (1) key design features, (2) disease area features, (3) trial operational challenges, (4) and results (where the study has been completed and data made accessible).

The second aspect of the analysis will focus on comparative analysis of different statistical methods that have been identified from the systematic review of statistical methods for basket trial design and/or analysis. Only methods with publicly available software will be included in the comparative analysis. The methods will be applied to real life basket trial data based on completed external studies where the data are available. The methods will also be applied on internal GSK data from past trials. The comparative analysis will examine the estimates of treatment effects and its uncertainties, impact of sample size, futility stopping rates and study conclusion (evidence of treatment effect or not).

[Note: the original protocol for the systematic reviews included three tables containing the number of hits from the PubMed/MEDLINE, Embase, and Cochrane Central Register of Controlled Trials. These tables are reported as Tables S1-S3 in Section 1 of this supplementary file, and we exclude the repetition of these tables here.]

## 9.3 Changes made to the systematic review protocol

The following changes were made to the original systematic review protocol:

1. For both the review of basket trial practice and basket trial methods, the systematic search for abstracts was completed only once for each of the three databases (i.e., PubMed/MEDLINE, Embase, and Cochrane Central Register of Controlled Trials Search criteria for both reviews). The search criteria reported in Tables S1-S3 were used instead of the criteria listed in Tables S7 and S8.
2. Due to differences in the extracted trial data between our systematic review of basket trial practice and the review of Park *et al*. [1], we extracted data from all identified basket trials (not just trials from 2019 to date).
3. During the data extraction process for the review of basket trial practice, we narrowed our definition of basket trials to include only trials that have a single treatment regimen. As a result, each of the 146 identified trials were open label without randomization or a control group.
4. Internal basket trial data from GSK was not used.

# 10. References

1. Park JJH, Siden E, Zoratti MJ, Dron L, Harari O, Singer J, Lester RT, Thorlund K, Mills EJ. Systematic review of basket trials, umbrella trials, and platform trials: a landscape analysis of master protocols. Trials (2019) 20: DOI:10.1186/s13063-019-3664-1 [PubMed Abstract](https://pubmed.ncbi.nlm.nih.gov/31533793/) | [CrossRef Full Text](https://www.ncbi.nlm.nih.gov/pmc/articles/PMC6751792/) | [Google Scholar](https://scholar.google.co.uk/scholar?hl=en&as_sdt=0%2C5&q=Park+et+al+2019+basket+trial+systematic+reveiw&btnG=)
2. Psioda MA, Xu J, Jiang Q, Ke C, Yang Z, Ibrahim JG. Bayesian adaptive basket trial design using model averaging. Biostatistics (2021) 22:19–34. DOI:10.1093/biostatistics/kxz014 [PubMed Abstract](https://pubmed.ncbi.nlm.nih.gov/31107534/) | [CrossRef Full Text](https://www.ncbi.nlm.nih.gov/pmc/articles/PMC7846150/) | [Google Scholar](https://scholar.google.co.uk/scholar?hl=en&as_sdt=0%2C5&q=Psioda+Bayesian+adaptive+basket+trial+design+using+model+averaging&btnG=)
3. Chu Y, Yuan Y. A Bayesian basket trial design using a calibrated Bayesian hierarchical model. Clinical Trials (2018) 15:149–158. DOI: 10.1177/1740774518755122 [PubMed Abstract](https://pubmed.ncbi.nlm.nih.gov/29499621/) | [CrossRef Full Text](https://www.ncbi.nlm.nih.gov/pmc/articles/PMC5891374/) | [Google Scholar](https://scholar.google.co.uk/scholar?hl=en&as_sdt=0%2C5&q=chu+et+al+A+Bayesian+basket+trial+design+using+a+calibrated+Bayesian+hierarchical+model&btnG=)
4. Park JJH, Hsu G, Siden EG, Thorlund K, Mills EJ. An overview of precision oncology basket and umbrella trials for clinicians. CA: A Cancer Journal for Clinicians (2020) 70:125–137. DOI:10.3322/caac.21600 [PubMed Abstract](https://pubmed.ncbi.nlm.nih.gov/32031692/) | [CrossRef Full Text](https://www.ncbi.nlm.nih.gov/pmc/articles/PMC7187272/) | [Google Scholar](https://scholar.google.co.uk/scholar?hl=en&as_sdt=0%2C5&q=Park+et+al+An+overview+of+precision+oncology+basket+and+umbrella+trials+for+clinicians&btnG=)
5. Hobbs BP, Pestana RC, Zabor EC, Kaizer AM, Hong DS. Basket Trials: Review of Current Practice and Innovations for Future Trials. Journal of Clinical Oncology (2022) 40:3520–3528. DOI:10.1200/jco.21.02285 [PubMed Abstract](https://pubmed.ncbi.nlm.nih.gov/35537102/) | [CrossRef Full Text](https://ascopubs.org/doi/10.1200/JCO.21.02285?url_ver=Z39.88-2003&rfr_id=ori:rid:crossref.org&rfr_dat=cr_pub%20%200pubmed) | [Google Scholar](https://scholar.google.co.uk/scholar?hl=en&as_sdt=0%2C5&q=Hobbs+et+al+2022+basket+trial+review&btnG=)
